# One‐Step Microfluidic Manufactured Fucose‐Decorated Sweetosomes Choose the Time and the Road for Their Intracellular Journey to Cancer Treatment

**DOI:** 10.1002/adhm.202505617

**Published:** 2026-05-13

**Authors:** Mattia Tiboni, Mariele Montanari, Shiva Khorshid, Michele Verboni, Andrea Duranti, Simone Lucarini, Daniele Lopez, Annalisa Aluigi, Gianluca Morganti, Michele Menotta, Giovanna Panza, Daniel J. Klionsky, Barbara Canonico, Luca Casettari

**Affiliations:** ^1^ Department of Biomolecular Sciences (DISB) University of Urbino Carlo Bo Urbino Italy; ^2^ Life Sciences Institute University of Michigan Ann Arbor Michigan USA

**Keywords:** active targeting, anticancer glycoscience, colorectal cancer (CRC) cells, endosomal escape, flow cytometry, liposomes, microfluidics

## Abstract

Carbohydrate‑functionalized liposomes are promising drug‑delivery systems due to their biocompatibility, biodegradability, low toxicity, and ability to mediate targeted cell interactions. However, conventional functionalization strategies rely on multi‑step chemical conjugations that introduce variability, hinder large‑scale production, and compromise formulation stability. Here, we overcome these limitations by achieving liposome functionalization without surface chemistry. We introduce “sweetosomes,” a newly formulated class of sugar‑decorated liposomes designed for cancer targeting, which promote more specific and active cellular uptake, prolonged intracellular retention, and enhanced endosomal escape. We show that organelle acidity can be selectively modulated by fucosylated sweetosomes, supporting their role in facilitating endosomal escape an essential step for the cytoplasmic delivery of biological therapeutics. Fucosylated sweetosomes and blank liposomes enter cells but traffic through distinct endosomal pathways; indeed, fucose residues appear to alter endosomal maturation and function. Our findings validate fucosylated sweetosomes as optimized lipid nanostructures for intestinal cancer targeting, demonstrating significantly improved curcumin delivery, primarily via the caveolae pathway. Finally, fucosylated sweetosomes highlight translational potential, due to their prolonged plasma persistence, as detected by ex vivo plasma and blood‑cell analyses.

## Graphical abstract

Sweetosomes, a new class of fucose‐decorated liposomes, are developed via a one‐step microfluidic process without surface chemistry. This study elucidates their main caveolae‐mediated entry and distinct endosomal trafficking. These nanostructures demonstrate superior endosomal escape, organelle acidity modulation, and prolonged plasma persistence, offering a robust and scalable translational platform for targeted cancer therapy.

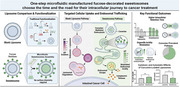

## 1 Introduction

Colorectal cancer (CRC) is the third most common cancer and a leading cause of cancer‐related deaths worldwide [1]. The human epithelial cell line Caco‐2 has long been used as an intestinal epithelial barrier model. Originally derived from colon carcinoma, this cell line, along with HT‐29, helps researchers study colon cancer biology, particularly in digestion and bioavailability studies due to its ability to express traits of mature intestinal cells. HT‐29 cells mimic intestinal goblet cells, which physiologically secrete mucins to facilitate bowel movement and protect the gut from irritants like food matrix components, reactive oxygen species (ROS), inflammatory mediators, and bacterial infections [2].

Given the overexpression of specific receptors on cancer cells, liposomal surfaces have been decorated with different ligands targeting these receptors. As sugars play a critical role in cancer biology, researchers are increasingly integrating glycoscience into anticancer therapeutic design [3, 4, 5]. Cell surfaces are rich in carbohydrates, such as glycolipids and glycoproteins, forming the glycocalyx, a potential target site for drug delivery. Despite limited research on carbohydrates as targeting agents, fucosylation has emerged as a key regulator of tumor cell proliferation and migration, influenced by the fucosyltransferase (FUT) 8 gene [6, 7]. Fucosylation, a posttranslational modification, has been identified as an indicator of diseases such as CRC, with L‐fucose demonstrating anticancer potential [8].

Carbohydrate‐functionalized liposomes offer a promising drug delivery system due to their biocompatibility, biodegradability, low toxicity, and targeted cell interaction [9]. Multiple carbohydrates, including mannose (Man), Man‐6‐phosphate, fucose, and galactose, have been conjugated to nano drug delivery systems (DDSs) to enhance receptor‐specific targeting [10]. Certain cancer cell lines actively internalize L‐fucose, secreting fucosylated proteins—indicating high L‐fucose demand in various tumors [11].

Fucosylated liposomes carrying anticancer drugs present a new strategy for CRC treatment, particularly in patients with advanced T‐factor staging, clinical progression, and metastasis [12, 13]. Unlike myeloid cells, intestinal cancer cell lines lack expression of MRC1/CD206 (mannose receptor C‐type 1) and CD209/DC‐SIGN (CD209 molecule), both involved in fucose binding [14, 15]. Nevertheless, while Caco‐2 and HT‐29 cells do not constitutively express classical myeloid C‐type lectins, their internalization capacity for fucosylated nano DDSs can be mediated by an alternative repertoire of epithelial lectins. Notably, these intestinal cancer cell lines exhibit high expression of Galectin‐3 [16, 17, 18] a lectin with a documented affinity for fucose‐containing glycans and Lewis‐type antigens [19, 20]. Furthermore, the presence of specific fucose‐binding sites confirms that lectins exhibit tropism for fucose residues. Therefore, fucose's high surface affinity on these cells [15] was leveraged in the present work to develop fucosylated sweetosomes, a newly formulated class of fucose‐functionalized liposomes designed specifically for intestinal cancer targeting and synthesized in one microfluidic step avoiding surface chemistry.

Surface functionalization is the bottleneck step in nanocarrier development because it typically requires complex surface chemistry, posing significant obstacles to reproducibility and scalability. Traditional methods involve multi‐step chemical conjugations that may introduce variability, hinder mass production, and complicate formulation stability. For the first time, this work overcomes these limitations by achieving sugar functionalization without surface chemistry. Instead, sweetosomes are formulated simply by mixing excipients/drug delivery vehicles within microfluidic devices, offering a reproducible, scalable, and efficient approach to cancer drug delivery. The neologism sweetosome would then consider a new class of sugar‐functionalized vesicles made using sugar fatty acid monoesters as active targeting excipients.

This paper details the step‐by‐step production and characterization of fucosylated sweetosomes, analyzing their cellular uptake, intracellular trafficking, and endosomal behavior. These factors are crucial in ensuring effective intracellular drug release, overcoming the endosomal/lysosomal barrier—an essential step for successful nanodrug delivery in cancer, neurodegenerative disorders, and infectious diseases [21]. Our findings validate fucosylated sweetosomes as optimized lipid nanostructures for intestinal cancer targeting, demonstrating increased curcumin delivery efficiency, mainly via the caveolae pathway. Additionally, we explore their endosomal fate, including their potential to be re‐released from cells after traversal through the endosomal pathway, while also considering their influence on glycomic profile and the expression of FUT8, a well‐known glycosylation‐related enzyme in CRC cells. Finally, ex vivo assessment in whole peripheral blood (both plasma and cells) highlights sweetosome differential internalization and striking translational aspects. Sensitive analytical tools such as flow cytometry (FC), confocal microscopy (CM), western blot (WB) and proteomic analysis (PA) were employed, alongside a cost‐effective protocol applicable to monitornew drug delivery systems (DDSs), particular nanomedicines [22].

The colloidal properties of sweetosomes were assessed via particle size analysis, polydispersity index (PDI), transmission electron microscopy (TEM) and cryo‐electron microscopy (Cryo‐EM) imaging. Further characterization was performed through Fourier‐transform infrared spectroscopy (FTIR).

## 2 Materials and Methods

### 2.1 Materials

Curcumin and palmitic acid were purchased from Tokyo Chemical Industry Europe (Belgium), 1,2‐dipalmitoyl‐*sn*‐glycero‐3‐phosphocholine (DPPC), 1,2‐dioleoyl‐*sn*‐glycero‐3‐phosphoethanolamine‐*N*‐(lissamine rhodamine B sulfonyl) (ammonium salt) (18:1 Liss Rhod PE), and cholesterol were obtained from Avanti Polar Lipids (USA). L‐fucose, triphenylphosphine, and di‐isopropyl azodicarboxylate/DIAD were purchased from Fluorochem (Hadfield, UK). The organic solvents at analytical grade were purchased from Merck‐Sigma Aldrich (Milan, Italy). 1,4‐dioxane was previously dried using MS 4Å. The correct structure of the synthesized L‐fucose palmitate was assessed by ESI‐MS and NMR analysis. ESI‐MS spectra were recorded with a Waters Micromass ZQ spectrometer in a positive or negative mode. NMR spectra were recorded via a Bruker AC 400 MHz spectrometer. Chemical shifts were measured by using the central peak of the solvent. Column chromatography purification was performed under “flash” conditions using Merck 230–400 mesh silica gel.

### 2.2 Procedure for the Synthesis of L‐Fucose Palmitate

A round‐bottom flask was charged with powdered L‐fucose (246 mg, 1.5 mmol, 1.5 equiv.) and anhydrous 1,4‐dioxane (20 mL, 0.05 M) under an argon atmosphere. After ultrasound irradiation of the resulting suspension for 15 min, palmitic acid (256 mg, 1.0 mmol, 1.0 equiv.) and Ph_3_P (787 mg, 3.0 mmol, 3.0 equiv.) were added to the mixture. Di‐isopropyl azodicarboxylate (0.591 mL, 3.0 mmol, 3.0 equiv.) was added dropwise to the stirred solution, and the resulting mixture was stirred vigorously at 20°C for 2 h. The reaction mixture was quenched with methanol, stirred for a further 5 min, and concentrated in vacuo. The resulting residue was purified by flash column chromatography (CH_2_Cl_2_:MeOH 99:1 to 95:5 v:v) to give L‐fucose palmitate 3 (270 mg, y = 67%) as a white solid.

^1^H NMR (400 MHz, MeOD) δ 0.89 (t, 3H, *J* = 7.0 Hz), 1.25 (d, 3H, *J* = 6.5 Hz), 1.38–1.28 (m, 24H), 1.61–1.67 (m, 2H), 2.46–2.32 (m, 2H), 3.52 (dd, 1H, *J* = 9.5, 3.5 Hz), 3.66–3.59 (m, 2H), 3.75 (qd, 1H, *J* = 6.5, 1.0 Hz), 5.39 (d, 1H, *J* = 8.0 Hz) ppm. ^13^C NMR (101 MHz, MeOD) δ 14.4, 16.6, 23.7, 25.7, 30.1, 30.4, 30.5, 30.6, 30.71, 30.76, 30.78, 30.79, 33.1, 35.0, 71.0, 72.8, 73.0, 75.1, 96.0, 174.3 ppm. ESI‐MS: 425 [M + Na]+, 447 [M+ HCOO]‐.

### 2.3 Formulation of Blank Liposomes and Fucosylated Sweetosomes by Microfluidics

The formulation of the liposomes and sweetosomes was achieved using an in‐house microfluidic device previously developed by 3D printing in our lab [23]. Briefly, the chip was made using polypropylene in a fused deposition modelling (FDM) 3D printer (Ultimaker 3, Ultimaker, the Netherlands), presenting a zigzag passive micromixing pattern that allows a complete mixing of the fluids in the channel. To prepare liposomes and sweetosomes, the lipidic excipients (i.e., DPPC, cholesterol, and fucose palmitate in sweetosomes) were dissolved in absolute ethanol together with curcumin when producing drug‐loaded carriers. The ethanolic solution was then pumped in the microfluidic device against MilliQ water at different flow rate ratios (FRRs), different total flow rates (TFRs), and different final lipid concentrations (mg/mL) as optimized previously [24] to modulate the final formulations’ characteristics (Table 1). The total flow rates were selected based on the performance previously obtained with our 3D‑printed microfluidic device. We chose a low TFR (8 mL/min), a high TFR (30 mL/min), and an intermediate value as a central point to investigate the impact of flow rate on the nanocarrier critical quality attributes. These conditions were identified as technically robust and representative of the practical operating window of the device.

**TABLE 1:** Microfluidic parameters to optimize the formulation of sweetosomes.

Parameter	F1	F2	F3
TFR (mL/min)	8	19	30
FRR (water:ethanol)	1:1	2:1	3:1
Final lipid conc (mg/mL)	5	10	15
Curcumin final concentration (mg/mL)	0.5	2.5	3.75

Sweetosomes were prepared using a 1:2:1 weight ratio of DPPC, cholesterol, and fucose palmitate, respectively as optimized previously [24]. Liposomes were prepared using a 3:1 weight ratio of DPPC and cholesterol, respectively, a final lipid concentration of 10 mg/mL a TFR of 19 mL/min, a FRR 2:1, and with or without curcumin at a final concentration of 2.5 mg/mL. To obtain fluorescent vesicles for in vitro studies, 1% w/w of rhodamine‐labeled lipid was added to the formulations.

### 2.4 Physicochemical Characterization of the Colloidal Systems

The characterization of the developed colloidal systems was conducted using multiple analytical techniques to ensure precise assessment of their physicochemical properties. The average particle size (Z‐average) and PDI were measured via dynamic light scattering (DLS) using a Malvern Zetasizer Nano S instrument (Malvern Instrument Ltd., UK), while the particles concentration (particles/mL) and the ζ‐potential was evaluated with the ZetaView x30 Twin (Particle Metrix, Germany).

The surface density of fucose on the vesicles was estimated for the selected formulation F2. The number of vesicles per milliliter was determined by nanoparticle tracking analysis (NTA), and the total amount of fucose palmitate present in the formulation was calculated from the known molar input. The corresponding number of fucose palmitate molecules was obtained using Avogadro's constant.

To derive an approximate ligand density, the total number of fucose palmitate molecules was divided by the number of vesicles measured by NTA. A correction factor of 0.5 was applied to account for the expected symmetric distribution of amphiphilic glycolipids between the inner and outer leaflets of the bilayer, providing an estimate of the number of fucose residues accessible on the vesicle surface (N according to the following equation:

$$N_{Fucose}=\frac{C_{FP}\times N_{A}\times 0.5}{MW_{FP}\times N_{vesicles}}$$

where C_FP_ is the concentration of fucose palmitate (g/mL), MW_FP_ is its molecular weight (402.57 g/mol), N_A_ is Avogadro's number (6.022 × 10^23^ mol^−1^), and N_vesicles_ is the vesicle concentration determined by NTA (particles/mL).

To further investigate vesicle morphology and size, TEM was performed using a Hitachi HT7800 (Hitachi, Japan) at an acceleration voltage of 100 kV. Negative staining was achieved with a 2% phosphotungstic acid solution in water. For sample preparation, a droplet of F2 formulation was deposited onto a formvar‐carbon‐coated copper grid, followed by the application of the staining solution after blotting with filter paper. The grid was then air‐dried overnight at room temperature after two washes with water.

Moreover, Cryo‐EM imaging was performed. For the preparation of the samples, vitrification was carried out with a Mark IV Vitrobot (Thermo Fisher Scientific). 3 µL of Liposomes and Sweetosomes (F2) samples (both at 10 mg/mL) in suspension in MilliQ water were added to a Quantifoil R 1.2/1.3 Cu 300‐mesh grids previously glow‐discharged at 30 mA for 30s in a GloQube (Quorum Technologies). 20 s after the application, the grids were blotted in a chamber at 4°C and 100% humidity and then plunge‐frozen into liquid ethane.

Vitrified grids were transferred to a Talos Arctica (Thermo Fisher Scientific) operated at 200 kV and equipped with a Ceta 16M detector (Thermo Fisher Scientific). Images were acquired at a nominal magnification of 45kx, corresponding to a pixel size of 0.229 nm/pixel with a defocus of 3 µm.

The scale bars represent 200 nm for the uncropped image and 100 nm for the cropped ones.

Additionally, attenuated total reflectance Fourier‐transform infrared spectroscopy (ATR‐FTIR, Spectrum Two, Perkin Elmer, USA) was employed to analyze the curcumin loaded F2 formulations alongside pure excipients and drug components, covering a spectral range of 400–4000 cm^−^^1^.

### 2.5 Stability Studies

The stability of the liposomes and fucosylated sweetosomes was studied for 30 days while the suspensions were maintained at 4°C or room temperature. Size PDI, and ζ‐potential were evaluated at the end of the study. In flow cytometry, a cationic lipophilic probe/LCD was used to evaluate membrane integrity, as well as the stability of rhodamine fluorescence after 30.

### 2.6 Drug Content Evaluation

The content of curcumin in the formulated suspension was evaluated using a UV spectrophotometer (Shimadzu UV‐1900i, Japan). The formulated liposomes and sweetosomes were dissolved in methanol by dilution, and the drug's content was studied at a wavelength of 413 nm. The content was obtained with a calibration curve of curcumin in methanol from 1 to 7.5 µg/mL and the loading degree was calculated as follows:

$$LD\%=\frac{Weight\;of\;drug\;in\;vesicles}{Total\;weight\;of\;vesicles}\;\times 100$$

### 2.7 Cell Uptake Studies on HT‐29 and Caco‐2

HT‐29 cells were cultured in RPMI 1640 Medium supplemented with 10% heat‐inactivated fetal bovine serum, 1% L‐glutamine (Sigma‐Aldrich, St Louis, MO, USA), and 1% penicillin‐streptomycin (Sigma‐Aldrich, St Louis, MO, USA) at 37°C in humidified air with 5% CO_2_. Caco‐2 cells were cultured in DMEM‐F12 medium supplemented with 10% heat‐inactivated FBS, 1% L‐glutamine, and 1% penicillin‐streptomycin at 37°C, 5% CO_2_. Both cell lines derived from intestinal cancer; Caco‐2 differentiates into absorptive cells, while HT‐29 develops into mucus‐secreting cells, the two primary cell types found in the intestine.

Rhodamine‐labelling was used to assess blank liposome (L) and sweetosome (S) uptake by intestinal cancer cells (HT‐29 and Caco‐2). Cancer cells were seeded in 6‐well plates (1.5 × 10^5^ cells/well) and treated with rhodamine‐labelled L and S (1:50 concentration) from 2 to 72 h. After incubation, vesicles were removed, cells detached by trypsinization, and washed with phosphate‐buffered saline/PBS at 260 g for 10 min. The uptake of the different vesicles in different intestinal cancer cell lines was determined by flow cytometry. The quantification and visualization of rhodamine‐labelled vesicles incorporation into cells was carried out using a FACSCanto II flow cytometer (BD, Franklin, Lakes, NJ, USA) equipped with an argon laser (blue, excitation 488 nm), a helium‐neon laser (red, excitation 633 nm), and a solid‐state diode laser (violet, excitation 405 nm) and a Leica TCS SP5 II confocal microscope (Leica Microsystem, Germany) with 488‐, 543‐, and 633‐nm illuminations and oil‐immersed objectives. In accordance with current standards for the study of extracellular vesicles (EVs) and synthetic vesicles, flow cytometry data are preferentially expressed as Mean or Median Fluorescence Intensity (MFI) rather than the percentage of positive cells [25, 26]. While percentage‐based quantification is suitable for binary, bimodal populations, it often lacks the sensitivity required to distinguish variations in the intracellular payload accumulation per cell [27]. This approach aligns with the MISEV guidelines, which emphasize the importance of reporting fluorescence intensity to ensure the reproducibility and quantitative rigor of uptake assays [28].

### 2.8 WB and Proteomic/Glycomic Profiles From L and S‐ Treated HT‐29 and Caco‐2 Cells

For WB, total proteins were extracted using the Protein Extraction Reagent Type 4 (Sigma–Aldrich). Cells were sonicated with 10 pulses of 10 s at 70W by a Labsonic 1510 Sonicator (Braun) and clarified by centrifugation for 10 min at 14000g. Protein concentration was determined by the Bio‐Rad Protein Assay, based on Bradford's method. Twenty micrograms of proteins were separated by SDS PAGE (Novex, Thermo Fisher Scientific), and then transferred to nitrocellulose (0.22 µm, Bio‐Rad). Membranes were probed with the primary antibody Anti FUT8 (ab198741, GTX17311, Prodotti Gianni) diluted in 5% w/v non‐fat dry milk in TBST. The utilized secondary antibody was HRP anti‐mouse or anti‐rabbit (Bio‐Rad). Immunoreactive bands were recorded using the enhanced chemiluminescence (Advansta) acquisition by ChemiDoc Touch Imaging System (Bio‐Rad). The whole lane normalization (WLN) strategy was adopted in western blot analyses. Acquired images were analysed by Image Lab software 5.2.1 (Bio‐Rad).

For Glycoproteomics, cells were directly processed by the EasyPep MS Sample Kit (Thermo Scientific Pierce). Peptides were desalted and dried with a Speed vacuum centrifuge (Savant‐SPD121P). Prior MS analysis they were solubilized in 0.1% formic acid and quantified by the quantitative colorimetric peptide assay (Thermo Fisher Scientific). Two micrograms of each sample were separated by the UltiMate 3000 RSLC nano system coupled to the Exploris 240 mass spectrometer (Thermo Fisher Scientific) and resolved by Easy‐Spray Pepmap RSLC C18 (2 µm, 75 cm × 75 µm) at 200 nL/min flow rate with a gradient of phase B (80% acetonitrile/0.1% formic acid, solvent A was 0.1% formic acid in water) from 2% to 40% in 150min. Phase B was then changed up 90% in 20 min, kept for 8 min, and then the column was re‐equilibrated for 20 min.

Data was acquired in positive mode and in data‐dependent manner. MS1 m/z range was set to 350–1500 at 120000 resolution (at m/z 200), charge state 2–8, AGC target 3^e6^, and auto maximum injection time. MS2 was adopted when ions intensity above 1^e4^, adopting first mass 110 m/z, normalized HCD collision energy in stepped mode 15%, 25%, and 35%, AGC target 2^e5^, and maximum injection time 100ms. The resolution was set to 30000 at m/z 200 and the internal calibrant was employed in run start mode. Analysis was performed in triplicate. Raw data generated by Xcalibur 4.2 software (Thermo Fisher Scientific) were analyzed using pGlyco3.1 [29]. Carbamidomethylation of cysteines was considered as a fixed modification, while as variable modifications the oxidation (M) and deamidation (N, Q). The precursor tolerance was set to 5ppm while fragment tolerance was 10ppm. Glycopeptide FRD was 0.01.

### 2.9 Cell Uptake Studies on Whole Blood Cells, in an Ex Vivo Model

The ex vivo cell uptake studies were conducted using fresh whole blood (WhB) obtained from healthy volunteers included in the Italian blood donor registry (A.V.I.S., Associazione Volontari Italiani Sangue), who provided signed informed consent. The blood samples were provided by the Santa Maria della Misericordia Hospital in Urbino. To evaluate the internalization efficiency, 500 µL aliquots of WhB were treated with blank liposomes (L) and sweetosomes (S) at a 1:50 dilution and incubated at room temperature. The uptake phase was evaluated at two different time points, 2 and 24 h, for evaluating interaction and the kinetic progression of the described nano delivery systems within the blood cells. Samples were then processed for immunophenotyping using a double staining approach to identify not only the main specific leukocyte subsets (Lympho, mono, and granulo) but also other specific subsets; by means of anti‐CD123 APC (clone 6H6) and anti ‐HLA‐DR FITC (clone G46‐6). The staining was performed for 15 min at room temperature (RT) in the dark. For the leukocyte analysis, RBCs were removed using a lysis buffer (in‐house NH_4_Cl‐based ACK buffer) for 10 min at RT. After centrifugation, the remaining white blood cell pellets were washed and resuspended in PBS for flow cytometric analysis. In parallel, to further investigate the interaction with specific mononuclear cell populations, Peripheral Blood Mononuclear Cells (PBMCs) were isolated from WhB through density gradient separation using Lymphoprep solution (Axis‐Shield PoC AS, Oslo, Norway). After isolation, PBMCs were washed twice in phosphate‐buffered saline (PBS) by centrifugation at 400 g and resuspended in complete medium (RPMI‐1640 supplemented with 10% v/v heat‐inactivated fetal bovine serum, 100 µg/mL penicillin, 100 µg/mL streptomycin, and 2 mM L‐glutamine). These isolated cells were then subjected to the same treatment and incubation protocols as described for WhB to compare the uptake profile in a purified environment. The uptake of L and S was quantified by measuring the Mean Fluorescence Intensity (MFI) across the gated populations, allowing for a comparative assessment of the internalization patterns between the two formulations over time (2 and 24 h). In parallel, the interaction of blank liposomes and sweetosomes with red blood cells (RBCs) was assessed by comparing their MFI against untreated control cells, while the presence and the relative concentration of L and S in plasma were quantitatively determined using Countbright Absolute Counting beads (Thermo Fisher Scientific, Waltham, MA, USA).

### 2.10 Compatibility Studies and the Impact on Intracellular Organelles

Viability assays were evaluated by flow cytometry‐based assays using 7‐aminoactinomycin D (7‐AAD, Beckman Coulter, USA) or propidium iodide/PI direct staining.

Autophagy determination was performed using an Autophagy Assay kit (ab139484; Abcam, Cambridge, UK) according to the manufacturer's protocol [30, 31, 32, 33]. The Autophagy Assay Kit ab139484 evaluates autophagic vacuoles and tracks autophagic flux in live cells by utilizing a dye that specifically labels these vacuoles. The green dye gathers in autophagic vacuoles depending on the pH levels within the vacuole. Additionally, it is pH stable for pre‐autophagosomes, autophagosomes, and autolysosomes. The mean fluorescence intensity (MFI) of the autophagy kit reflects the degree of autophagy in the cell population. For flow cytometry, cells were incubated with 300 µL of the diluted green stain solution for 30 min at 37°C in the dark after treatment. Acidic lysosomes and the pH of lysosome‐related organelles were assessed using LysoTracker Deep Red (Thermo Fisher Scientific, L12492) and LysoSensor Green DND‐189 (Thermo Fisher Scientific, L7535), respectively. At 37°C for 30 min, cells were labeled with either 100 nM LysoTracker or 500 nM LysoSensor. LysoTracker and LysoSensor signals were evaluated by flow cytometry or confocal microscopy [34]. The creation of ROS was evaluated by the cytometric test of cells labelled with 5‐(and 6‐)chloromethyl‐2’,7’‐dichlorodihydrofluorescein diacetate acetyl ester (CM‐H_2_DCFDA, Thermo Fisher Scientific, USA), which can detect intracellular H_2_O_2_ production. The amount of peroxide created determines the intensity of DCF fluorescence within cells. CM‐H_2_DCFDA was utilized at a final concentration of 5 µm for 30 min at 37°C [35].

### 2.11 Intracellular Detection of CD63/Tetraspanin‐30/ LAMP3, CD107a/LAMP1, and CD81/Tetraspanin 28

The intracellular antigen distribution required a technique that employs cell fixation, permeabilization, and staining. Intracellular markers were evaluated using fluorescent antibodies. After short and longer L and S uptake, the cells were trypsinized at 37°C for a few minutes, fixed in 4% paraformaldehyde for 15 min at room temperature, suspended in 500 µL of phosphate‐buffered saline and maintained for one week at 4°C. Before the analysis, cells were centrifuged at 400 g for 5 min, and pellets were resuspended in 300 µL Cytoperm reagent. Cells were stained with monoclonal anti‐human antibodies anti‐CD63 (Clone H5C6, SONY) Alexa Fluor 488 conjugated, anti‐CD81 (Clone JS‐81) FITC conjugated, or anti‐LAMP1/CD107a (Clone H4A3) APC conjugated according to the manufacturer's instructions.

### 2.12 Pulse‐Chase Experiments: Cell and Media Analyses

Caco‐2 and HT‐29 cells were incubated with rhodamine‐labelled vesicles for 2 h; after this treatment, the media were removed, the cells were washed, and the media renewed. The first batch of untreated and liposome‐treated Caco‐2 and HT‐29 cells were acquired by FC, together with the respective 2‐h dilution medium (i.e., the amount of liposome added in each well, in starting the experiment except the amount internalized by the cells). Cells (from the other experimental batches) were incubated in fresh media for 4 and 24 h from the initial treatment, and they were harvested and analyzed by FC. The MFI values of the cells analyzed at 4 and 24 h were compared to those of cells that were pulsed for 2 h, which was considered 100%. For each acquisition, dead cells and cell doublets were excluded by setting the gates in the forward scattering and side scattering plots, and for each sample at least 10000 cells were acquired. Counting beads were added (sample:beads ratio 1:1) to the sample media for total liposome counts, and at least 20000 events were acquired. According to nanoparticles and/or extracellular vesicle (EV) FC detection, forward scatter (FSC) and side scatter (SSC) parameters were in log visualization, and the instrument was set using Rosetta beads. The threshold was set up on the FL2 channel due to the emission of rhodamine‐labelled liposomes.

### 2.13 Free L‐fucose Treatment for Specific Inhibition of Uptake

To evaluate the internalization efficiency, HT‐29 and Caco‐2 cells were treated with L and S at a 1:50 dilution, monitoring uptake at 2 and 24 h. A competitive inhibition assay was performed by pre‐treating the cells with an excess of free L‐fucose at a final concentration of 50 mm for 40 min. This treatment was used to evaluate the potential inhibition of binding and uptake of Liposomes and Sweetosomes, specifically involving fucose‐dependent recognition pathways, similarly to several authors [36, 37]. After the incubation time course, the cells were washed with PBS to remove non‐internalized L and S and detached for flow cytometric analysis. L and S uptake, both in the presence and absence of the fucose inhibitor, was quantified by measuring the MFI.

### 2.14 Endocytosis‐Specific Inhibition

Different inhibitors were employed to evaluate which pathways (or combination of pathways) are involved in sweetosome uptake and internalization and the specific advantage (if any) concerning undecorated liposomes. In detail, cells were pretreated with the following inhibitors: 4°C (low temperature), chlorpromazine hydrochloride, nocodazole, genistein, EIPA, dynasore or hypertonic sucrose, followed by the addition of L and S for 2 h, then sample analysis by FC. Negative controls (i.e., cells without the presence of liposomes) were also carried out. Concentrations, time, and pathways involved are reported in Table 2.

**TABLE 2:** Endocytosis inhibitors used in the study.

Inhibitor	Time of pretreatment (min)	Concentration	Endocytosis pathway addressed	Mechanism
+4°C	30		Universal inhibitor that suppresses the process of endocytosis and exocytosis	Energy‐dependent process inhibitors [38]
Chlorpromazine hydrochloride	30	5 µg/mL	Clathrin‐mediated endocytosis inhibitor	AP2 inhibitor; blocks endosome recycling [39]
Nocodazole	30	7.5 µg/mL	Macropinocytosis inhibitor	Microtubule assembly inhibitor [40, 41]
Genistein	30	20 µg/mL	Caveolae‐mediated endocytosis inhibitor	Several tyrosine kinases inhibited [42, 43]
EIPA	30	50 µM	Macropinocytosis inhibitor	Na^+^/H^+^ exchanger pump inhibitor [44]
Dynasore	30	80 µM	Clathrin‐mediated and caveolae‐mediated endocytosis inhibitor	Dynamin GTPase inhibitor [45, 46]
Hypertonic Sucrose	60	300 mM	Clathrin‐mediated endocytosis inhibitor	Formation of type 1 coated pits inhibitor [47]

### 2.15 Release Efficiency of Encapsulated Curcumin and Evaluation of Antiproliferative and Cytotoxic Effects

Absolute cell counting was performed using Countbright Absolute Counting beads (Thermo Fisher Scientific, Waltham, MA, USA). Approximately 20000 cell/events were collected. The setup and calibration procedures were optimized for the absolute counting protocols. Cell death was evaluated using 7‐AAD (Beckman Coulter, USA); no permeabilized cells were incubated for 15 min in the dark. Intracellular reactive oxygen species (ROS) content was measured with 5 µM of CM‐H2DCFDA, Thermo Fisher Scientific, USA) incubated for 20 min. To evaluate changes in cell morphology, both cytometry and an inverted light field microscope were used. In flow cytometry, populations that differed in granularity were distinguished using their physical characteristics, side scatter (SSC). The cells were observed under a bright‐field inverted light microscope Nikon Eclipse TS100 (Nikon, Japan) at 40× magnification, to highlight the detachment of cells, cell density, and morphological changes related to cell death.

### 2.16 Statistical Analysis

Quantitative data are expressed as the mean ± SD based on at least three independent experiments. Analysis of variance (ANOVA) approaches were used to compare values among more than two different experimental groups for data that met the normality assumption. One‐way ANOVA or two‐way ANOVA were followed by a Tukey or Dunnet post‐hoc test. The means of the two groups were compared by using an unpaired *t*‐test. For FUT8 expression analysis, the non‐parametric Friedman test was used, while glycomics data were analyzed using Brown‐Forsythe and Welch ANOVA tests. A *p*‐value < 0.05 was considered significant. All statistical analyses were performed using GraphPad Prism 9.0 (GraphPad Software, San Diego, CA, USA).

## 3 Results and Discussion

### 3.1 Microfluidic Formulation and Characterization of Sweetosomes

Microfluidics represents a state‐of‐the‐art platform for the scalable and tunable production of nanomedicines [48]. In this study, we report an advancement of the one‐step microfluidic synthesis of a new class of sugar decorated vesicles, that we called sweetosomes, achieved without any post‐synthetic surface modification. By co‐formulating phospholipids and fucose palmitate under controlled flow conditions, we enabled the direct and homogeneous surface presentation of fucose residues. This streamlined approach enhances reproducibility, simplifies manufacturing, and opens new avenues for targeted nanotherapeutics.

Moreover, with the aim to obtain a scalable formulation, the microfluidic synthesis allows to operate under continuous‑flow conditions, meaning that the maximum batch volume is theoretically unlimited as long as reagent solutions are supplied. In this context, production scale is determined by the duration of operation rather than by the physical volume of a reaction vessel. Furthermore, the 3D‑printed microfluidic chip used in this study can be easily parallelized to increase throughput, and its low fabrication cost allows either repeated use after appropriate cleaning [49] or single‑use operation when sterility or cross‑contamination concerns arise. The physicochemical attributes obtained at the small volumes used in this R&D phase are maintained when increasing production volume, since the microfluidic method and operating parameters remain unchanged. This supports the intrinsic scalability and reproducibility of the process.

The fucose derivatization was obtained using fucose palmitate. It belongs to the class of sugar monoesters and was specifically synthesized for this work as reported in the method section. The glycosylated architecture of sweetosomes aims to promote active drug internalization through transporter‐mediated pathways while mitigating cytotoxicity, allowing a potential application as a cancer‐targeted delivery system.

The optimum ratio between commercial sugar esters and lipids was optimized in our previous work [24], so here, we focus on the optimization of the final characteristics of the vesicles using our 3D printed microfluidic device.

In Table 3 below, the results in terms of size, PDI, ζ‐potential, and loading degree (LD%) are reported for the three microfluidic conditions tested.

**TABLE 3:** Physicochemical characterization of the microfluidic conditions tested to formulate sweetosomes.

Sweetosomes	Size (nm)	PDI	ζ‐potential (mV)	Curcumin LD%
F1	154 ± 7	0.154 ± 0.028	−3.13 ± 0.04	7.6 ± 0.2%
F2	145 ± 7	0.190 ± 0.040	−2.29 ± 0.26	10.5 ± 0.1%
F3	167 ± 15	0.250 ± 0.016	−3.01 ± 0.09	9.3 ± 0.5%

Based on the reported results, the formulation F2 having the smallest size, a narrow PDI <0.25, and the highest LD% was selected to continue the studies. Drug free sweetosomes formulated with the same microfluidic parameters of F2 presented a size of 123 ± 2 nm, PDI 0.071 ± 0.006, −4.2 ± 0.3 mV of ζ‐potential. Simultaneously, blank liposomes reported a size of 135 ± 2 nm, a PDI of 0.083 ± 0.005, and a ζ‐potential of −7.7 ± 0.2 mV with a slight increase when curcumin was loaded obtaining a curcumin‐loaded liposome of 156 ± 3nm, PDI 0.114 ± 0.003, −5.5 ± 0.7 mV of ζ‐potential, and a LD% of 9.8 ± 0.2%.

After 30 days of storage, both drug‐free liposomes and fucosylated sweetosomes maintained good physicochemical stability at 4°C and room temperature, with only minor variations in size, dispersity, and surface charge. Liposomes stored at 4°C showed a mean diameter of 156 ± 1 nm, PDI 0.104 ± 0.006, and ζ‑potential −6.4 ± 0.2 mV, while those kept at room temperature showed a mean diameter of 145 ± 2 nm and a PDI 0.049 ± 0.005, with a comparable ζ‑potential (−7.1 ± 0.1 mV). Fucosylated sweetosomes exhibited a similar modest increasing trend: at 4°C they measured 145 ± 5 nm with PDI 0.103 ± 0.019 and ζ‑potential −5.1 ± 0.08 mV, whereas room‑temperature samples showed a size of 134 ± 3 nm keeping a good homogeneity (PDI 0.085 ± 0.012), with a ζ‑potential of −4.7 ± 0.2 mV. Overall, the limited changes across conditions suggest that both nanocarrier types retain structural integrity and colloidal stability over one month of storage.

The surface density of fucose on the vesicles was then estimated for this formulation using a stoichiometric approach based on the known molar amount of fucose‑palmitate added to the formulation and the number of vesicles measured by NTA using the equation reported in the method section. Using the known fucose palmitate concentration in F2 (2.5 mg/mL), its molecular weight (402.57 g/mol), the vesicle concentration measured by NTA (1.3 × 10^1^ ^3^ particles/mL), and assuming symmetric distribution of the glycolipid between the two leaflets, the estimated surface density corresponded to approximately 1.4 × 10^5^ fucose residues per vesicle. This method is inherently semi‑quantitative, as it does not directly measure the fraction of fucose‑palmitate effectively incorporated into the outer leaflet, nor does it account for potential differences in incorporation efficiency among formulations. Nevertheless, it provides a reasonable indication of the order of magnitude of surface functionalization for F2.

**FIGURE 1:**
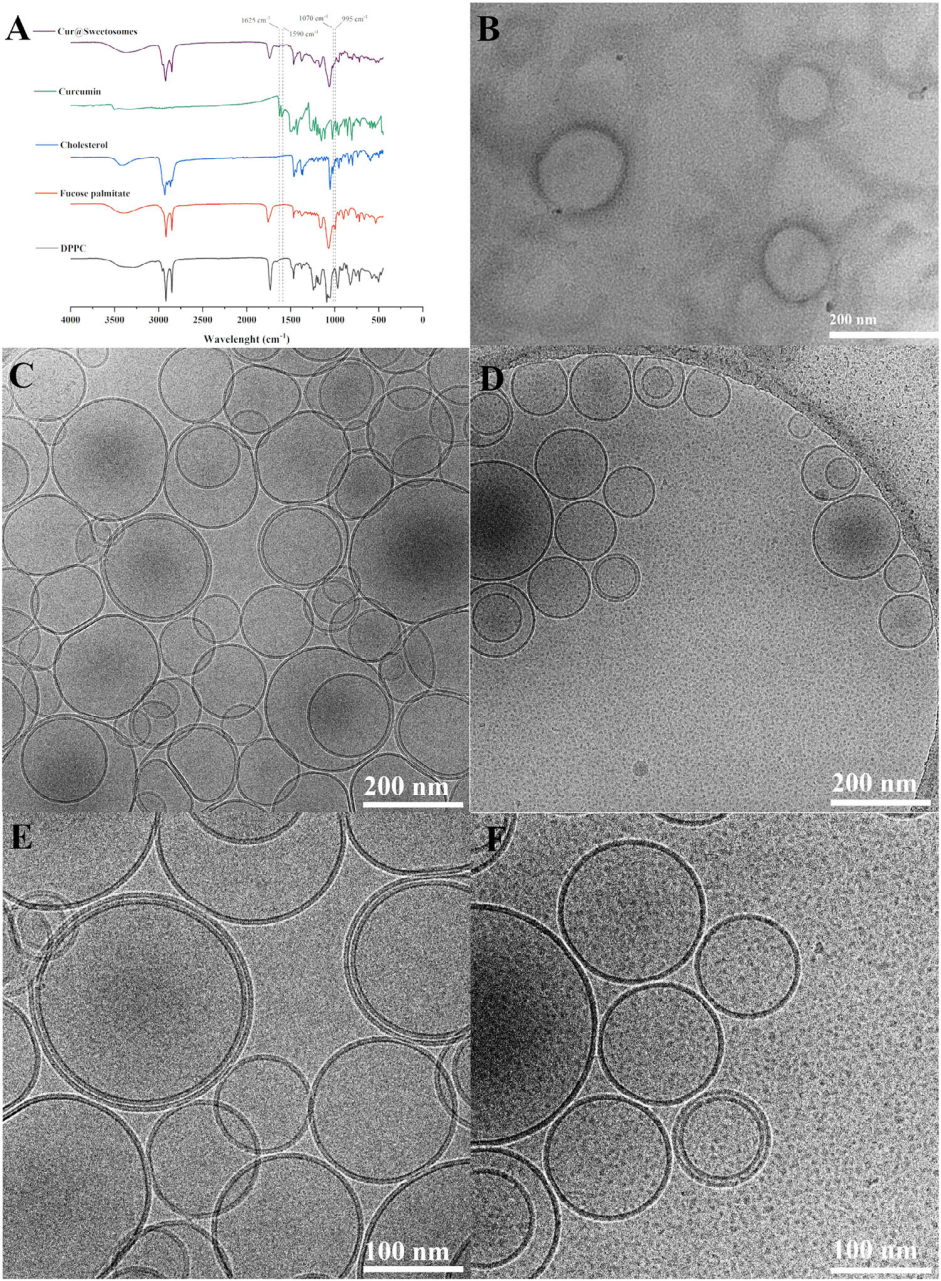
Sweetosomes characterization: (A) FTIR Analysis of curcumin‐loaded sweetosomes; (B) TEM Sweetosomes; (C) Cryo‐EM Liposomes; (D) Cryo‐EM Sweetosomes; (E) Cryo‐EM Liposomes (Magnification); (F) Cryo‐EM Sweetosomes (Magnification).

To further characterize the curcumin‐loaded sweetosomes, FTIR analysis was performed, and the results were reported in Figure 1A. From the obtained FTIR spectra, peaks at 1625 and 1590 cm^−1^ in the curcumin were assigned to C═O stretching of the β‐diketone moiety and C═C stretching in the aromatic rings and conjugated system, respectively [50]. In the cholesterol spectrum, the characteristic peaks can be seen at 3405, 2866, 1466, 1376, and 1056 cm^−1^, which refers to stretching hydroxyl group, symmetric C─H stretching vibration, asymmetric stretching vibration of methylene and methyl group, bending vibration of methylene and methyl group, and bending vibration of C─O group, respectively [24]. In the fucose palmitate spectrum, the peak at 1756 cm^−1^ corresponds to the C═O stretching of an α,β‐unsaturated ester, while the bands at 1070 and 995 cm^−1^ are attributed to C─O stretching and C─O─C ring vibrations of the fucose moiety, respectively. In the sweetosome's spectrum, the peak at around 3350 cm^−1^ was related to cholesterol meanwhile peaks at 1625 and 1590 cm^−1^ were assigned to the presence of the active molecule, and peaks at 1070 and 995 cm^−1^ to the presence of the sugar ester.

TEM visualization (Figure 1B) showed a round‐shaped morphology and confirmed the size and the narrow polydispersity. Cryo‑TEM analysis of liposomes and sweetosomes (Figure 1C–F) provided direct visualization of the vesicle architecture and consistently showed a homogeneous population of spherical, unilamellar vesicles across the imaged fields. Only a small subset of particles showed multilamellar or compartmentalized structures.

### 3.2 The Uptake of Empty, Blank Liposomes and Sweetosomes is Dissimilar in Time and Cell Specificity, Induces Negligible Effects on Cell Viability, and Differentially Involves the Endolysosomal Network While not Significantly Impacting Glycomic and Cell Cycle Profiles

**FIGURE 2:**
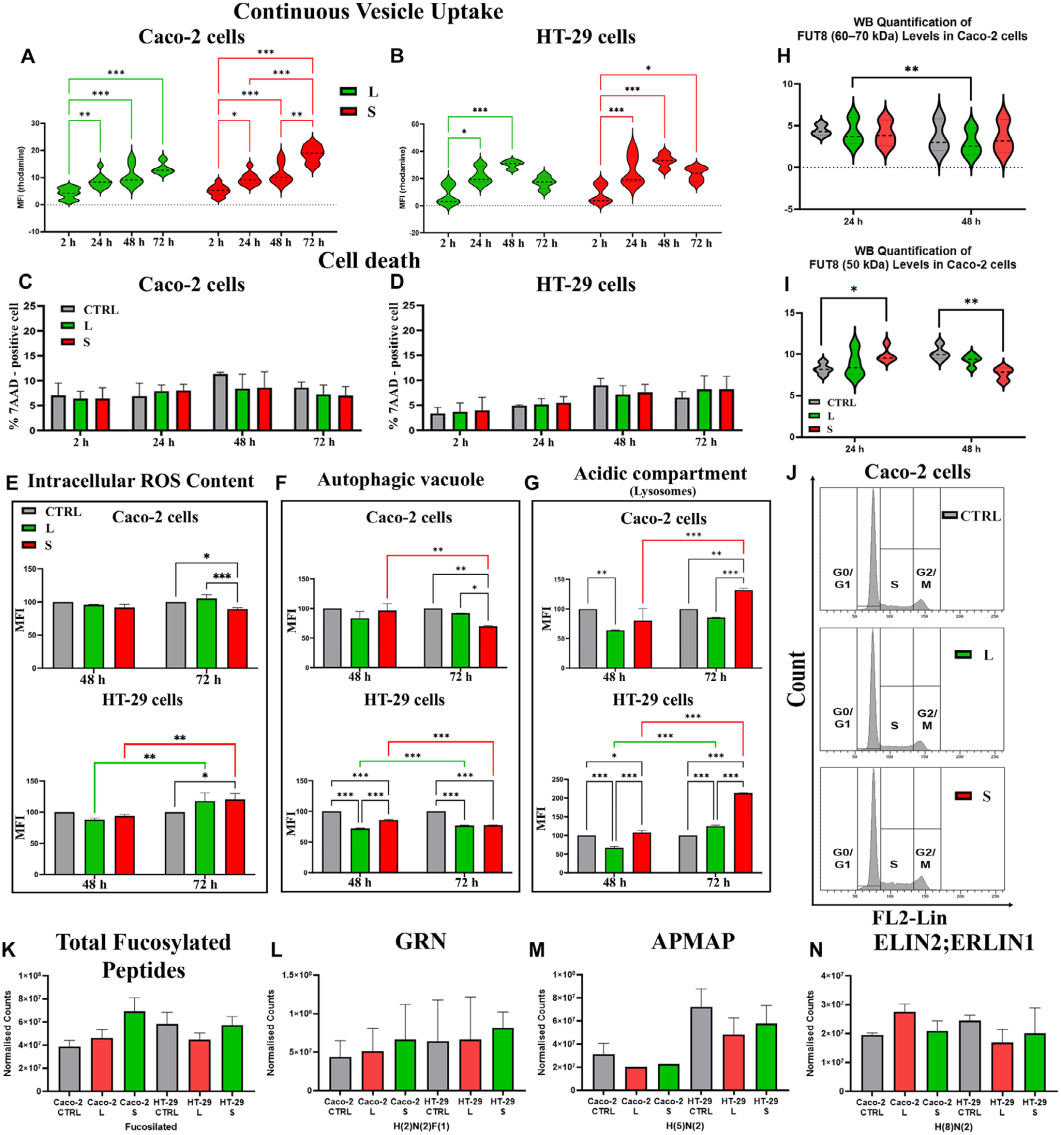
Sweetosome targeting strategy do not promote tumor progression: continuous exposure reveals an increase of sweetosomes uptake, affecting endolysosome and autophagosome networks without any significant cell death induction and alteration of glycomic and cell cycle profiles. Subcellular effects induced by liposomes (L) and sweetosomes (S) on two intestinal cancer cell lines. (A,B) L (green histograms) and S (red histograms) uptake by Caco‐2 (A) and HT‐29 (B) cells from 2 h to 72 h. (C,D) Cell death was analyzed as the relative percentage of cell positivity for 7‐AAD in Caco‐2 (C) and HT‐29 (D) cells from 2 to 72 h. (E) The DCF MFI evaluated the intracellular ROS level in Caco‐2 cells (above) and HT‐29 cells (below) for 48 and 72 h. (F) The autophagic vacuoles are presented as MFI of the green dye from the Autophagy Kit in Caco‐2 cells (above) and HT‐29 cells (below) for 48 and 72 h. (G) The LysoTracker Deep Red (LTDR) MFI investigated the lysosomal compartment in Caco‐2 cells (above) and HT‐29 cells (below) for 48 and 72 h. (H,I) WB quantification of FUT8 levels (60–70 kDa and 50 kDa, respectively) in Caco‐2 cells at 24 and 48 h. (J) Representative cell cycle profiles of Caco‐2 cells at 48 h. (K–N) Evaluation by glycoproteomics at 48 h in Caco‐2 and HT‐29 cells of the relative abundance of total fucosylated peptides (K) and specifically of GRN (L), APMAP (M), and ERLIN2;ERLIN1 (N).

The two cell lines employed in this study, Caco‐2 and HT‐29 cells, represent human colorectal cancer lines with peculiar differences [51, 52, 53], as discussed above. These differences included the cell doubling time, corresponding to 70–80 h for Caco‐2 cells and about 20 h for HT‐29 cells. As expected, these differences affect the continuous liposome uptake, as particularly highlighted by MFI values (Figure 2A,B). While the percentage of internalizing cells (Figures S1A,B and S2) shows an initial increase after 2 h, it reaches a plateau (at 24/48 h) that persists until 72 h, suggesting a steady‐state population. However, this approach is relatively insensitive to the intracellular fate of the carriers. Unlike percentage‐based data, MFI analysis provides the necessary resolution to track the onset of liposome degradation or exit processes, offering a more dynamic picture of the internalization kinetics over time. By MFI, liposomes and sweetosomes displayed almost the same degree of uptake in Caco‐2 tumoral cells from 0 to 48 h, slightly increasing during this time‐course. In contrast, after 72 h, an increase was observed in MFI values for sweetosomes. After 24 h, rhodamine fluorescence in HT‐29 cells showed a more relevant continuous uptake for sweetosomes.

After 48 h, the similar internalization levels of the two liposomal preparations can be attributed to cell proliferation processes. Specifically, the twofold increase in MFI observed at 24 h did not further escalate at 48 h, as active cell division resulted in the dilution of cellular components between daughter cells. Notably, after 72 h of continuous interaction of both formulations with HT‐29 cells, the rhodamine MFI decreased, revealing a significantly higher uptake for sweetosomes. Furthermore, the impact of both empty formulations on cell viability was assessed from 2 to 72 h; however, no significant increase in cell death was observed for either treatment in the tested cell lines (Figure 2C,D). Nevertheless, other cell compartments are affected differently in the two cell lines. For example, sweetosomes resulted in a decrease in ROS levels, mainly H_2_O_2_, after 72 h in Caco‐2 cells (Figure 2E). In contrast, HT‐29 cells displayed a similar ROS increase for both formulations after 72 h. Indeed, given that targeting endolysosomal compartments represents a new therapeutic strategy to fight cancer and also overcome multidrug resistance/MDR, organelles of the endocytic‐autophagic pathway have been investigated; these processes can be triggered by drugs directly or by a combination of different drugs and the specific DDS employed [54, 55, 56]. Sweetosome uptake lowered the amount of autophagic vacuoles in Caco‐2 cells after 72 h, whereas liposomes had no effect (Figure 2F). Finally, lysosome acidification was higher after 72 h than after 48 h, in both tumor cell lines (Figure 2G). Such a framework suggests the need to study exit processes, as well as specific endocytic pathways, along with internalization. While characterizing the intracellular fate of these carriers is essential, the functional consequences of ligand internalization also warrant careful investigation: a potential concern regarding fucosylated delivery systems is whether the internalized fucose could provide a metabolic advantage to cancer cells. Notably, the fucose moiety appears not to gain the aggressive phenotype of intestinal cancer cells. Specifically, Western blot analysis, revealed that FUT8 (primarily synthesized as a type II transmembrane protein of approximately 66–70 kDa, [57] exhibit an expression and activity similar to control levels following S treatment (Figure 2H), suggesting no significant induction of the core‐fucosylation pathway: only a modest although significant decrease is observed at 48h after blank liposome addition. Indeed, FUT8 frequently undergoes proteolytic cleavage within its stem region, a process that releases an enzymatically active soluble form of approximately 50 kDa [58, 59]: this 50 kDa fragment corresponds to the C‐terminal catalytic domain and is frequently detected in colorectal cancer (CRC) models, including Caco‐2 cells [60]. The relative profiles are reported in Figure 2I, demonstrating that the treatment of Caco‐2 cells with sweetosomes does not induce a pro‐tumorigenic upregulation of core‐fucosylation pathways. In fact, while the levels of the mature 66 kDa FUT8 isoform remained stable, the 50 kDa isoform exhibited a transient increase at 24 h, followed by a significant downregulation below control levels at 48 h. Furthermore, no increase in cell proliferation was observed (Figure 2J) further confirming that the exogenous fucose pool provided by sweetosomes is insufficient to permanently increase FUT8 and to trigger pro‐survival signaling or metabolic shifts.

Moreover, total *N*‐fucosylated peptides and specifically Progranulin (GRN) were evaluated (Figure 2K,L). GRN is a highly glycosylated protein that traffics through the endolysosomal pathway, localizing within endosomes and late endosomes to regulate lysosomal homeostasis, enzymatic activity, and protein degradation [61, 62]. In addition, to generally monitor glycomic profiles the following proteins: APMAP, and ERLIN1/2 (critical modulators of the tumor microenvironment by regulating epithelial‐mesenchymal transition, l and ER‐stress‐induced survival of malignant cells) were also evaluated. These data reveal a substantial preservation of core *N*‐fucosylation and general glycosylation across both control samples and those treated with either delivery formulations (Figure 2K–N).

### 3.3 Pulse‐Chase Experiments Make it Possible to Distinguish Both Formulations’ Internalization and Persistence in the Two Colon Cancer Cell Lines, Without Interference From the Competing Exit Processes: Impact on Targeting and Penetration

**FIGURE 3:**
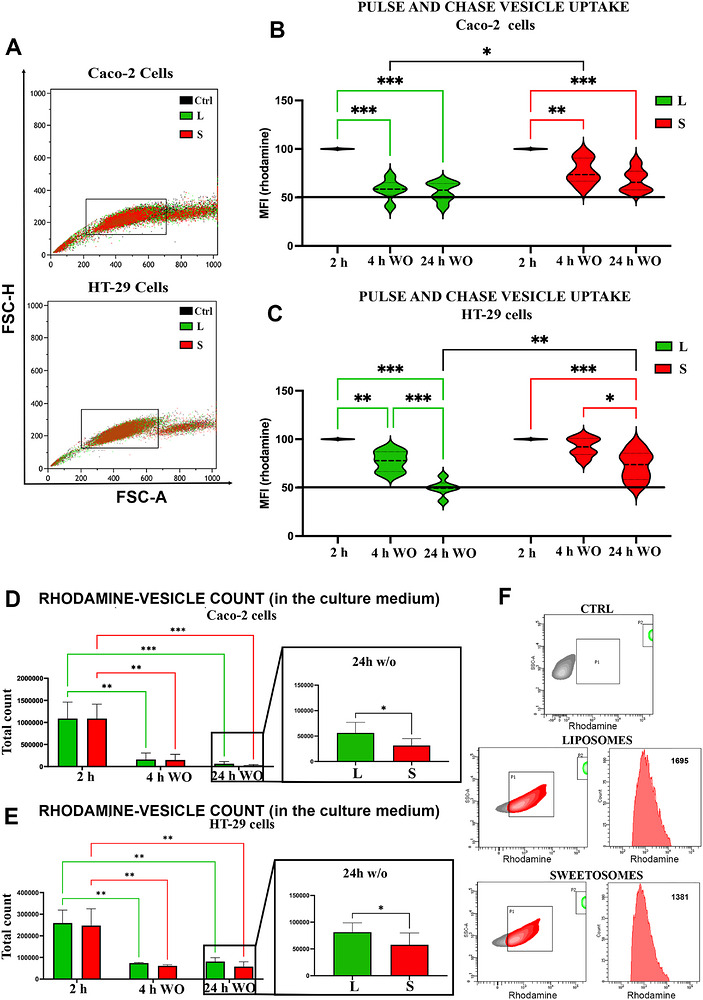
A two‐h pulse followed by a 4‐ and 24‐h chase reveals stronger sweetosome permanency, partially explained by reduced liposome release. (A) Overlay dot plots show the gating strategy to analyze light scatter parameters for the Ctrl (black dots), L (green dots), and S (red dots) in Caco‐2 (above) and HT‐29 (below) cells to exclude doublets or replicating cells. (B,C) Violin plots and statistical analysis of rhodamine fluorescence permanency inside Caco‐2 (B) and HT‐29 (C) cells after 2‐h exposure to rhodamine‐labelled liposomes (pulse) at different time points of chase (4 and 24 h). (D,E) Statistical histogram of the total count of L and S presence in Caco‐2 (D) and HT‐29 (E) medium after 2‐h exposure to rhodamine‐labelled liposomes (pulse) at different time points of chase (4 and 24 h). (F) The gating strategy to analyze the total count of L and S in the culture medium: red corresponds to the population of interest (rhodamine‐positive events), green to the counting beads, and gray to rhodamine‐negative events. The histograms represent the difference between the formulations in rhodamine MFI, also reported in. For each condition, two independent replicate samples were measured, and at least 10 000 cells were acquired for each sample.

To dissect the delicate balance between cellular uptake and exit kinetics, we performed “pulse and chase” experiments [63] on both cell lines. This approach is crucial because, under continuous exposure, intracellular fluorescence is never a simple measure of uptake; rather, it represents a dynamic equilibrium influenced by competing “exit” factors such as cell division, drug delivery system degradation, exocytosis, or the leakage of the fluorescent probe. Caco‐2 and HT‐29 cells were treated with rhodamine‐labelled liposomes or sweetosomes for 2 h, after which the treatment medium was removed, the cells washed, and the medium renewed. Cells were then incubated in fresh medium for 4 and 24 h without formulations (4 h wo and 24 h wo), and they were harvested and analyzed by flow cytometry to quantify red fluorescence. Dead cells and cell doublets were excluded by setting the gates in the forward scattering (FSC) FSC‐A vs FSC‐H plots (Figure 3A). The “chase” fluorescence values from the cells were compared to those of cells that were pulsed for 2 h without a chase (Figure 3). These data highlight a longer intracellular persistence for sweetosomes in both colorectal cancer cell lines, characterized by a distinct time modulation. In Caco‐2 cells, a fluorescence drop was detected starting at 4 h (Figure 3B); notably, between the processes degradation and release of blank liposome, the former appears prevalent, as demonstrated by the minimal amount of red fluorescent events detected in the medium. This fluorescence value was maintained at almost the same level for 24 h, presumably due to the unusual doubling time (60‐70 h) of Caco‐2 cells that in 24 h cannot further dilute the fluorescence. Although sweetosomes showed a similar trend, they exhibited a decrease in MFI of about 25%–30%, compared to the 50% decrease of rhodamine MFI registered for blank liposomes. Indeed, depending on the mean doubling time for the HT‐29 cell line, rhodamine MFI particularly decreased for blank liposomes after 24 h, with a significant reduction (about 30%) starting after 4 h from treatment (Figure 3C). By comparison, this fluorescence drop was lacking for sweetosomes and appeared only after 24 h of chase. We can assume that liposomes, after 4 h from their administration, were mainly degraded, because the counts of rhodamine‐labeled events (Figure 3D,E), as detected by FC and bead addition (Figure 3F), did not reveal substantially greater levels of release, compared to those observed for sweetosomes. After 24 h, for both blank liposomes and sweetosomes such “exit processes” seem to contribute to fluorescence loss but to different extents. To strengthen the reliability and meaning of these data, specific MFI values of each formulation were evaluated, revealing a different rhodamine MFI in starting experiments for both blank liposomes and sweetosomes (Figure S3). and their stability over time (Figure S4).

### 3.4 Free L‐Fucose Treatment Underscores the Role of Fucose Moieties of Sweetosomes: Their Preferential Binding and Internalization are, at Least Partially, Derived by Their Engagement, as Multivalent Ligands

**FIGURE 4:**
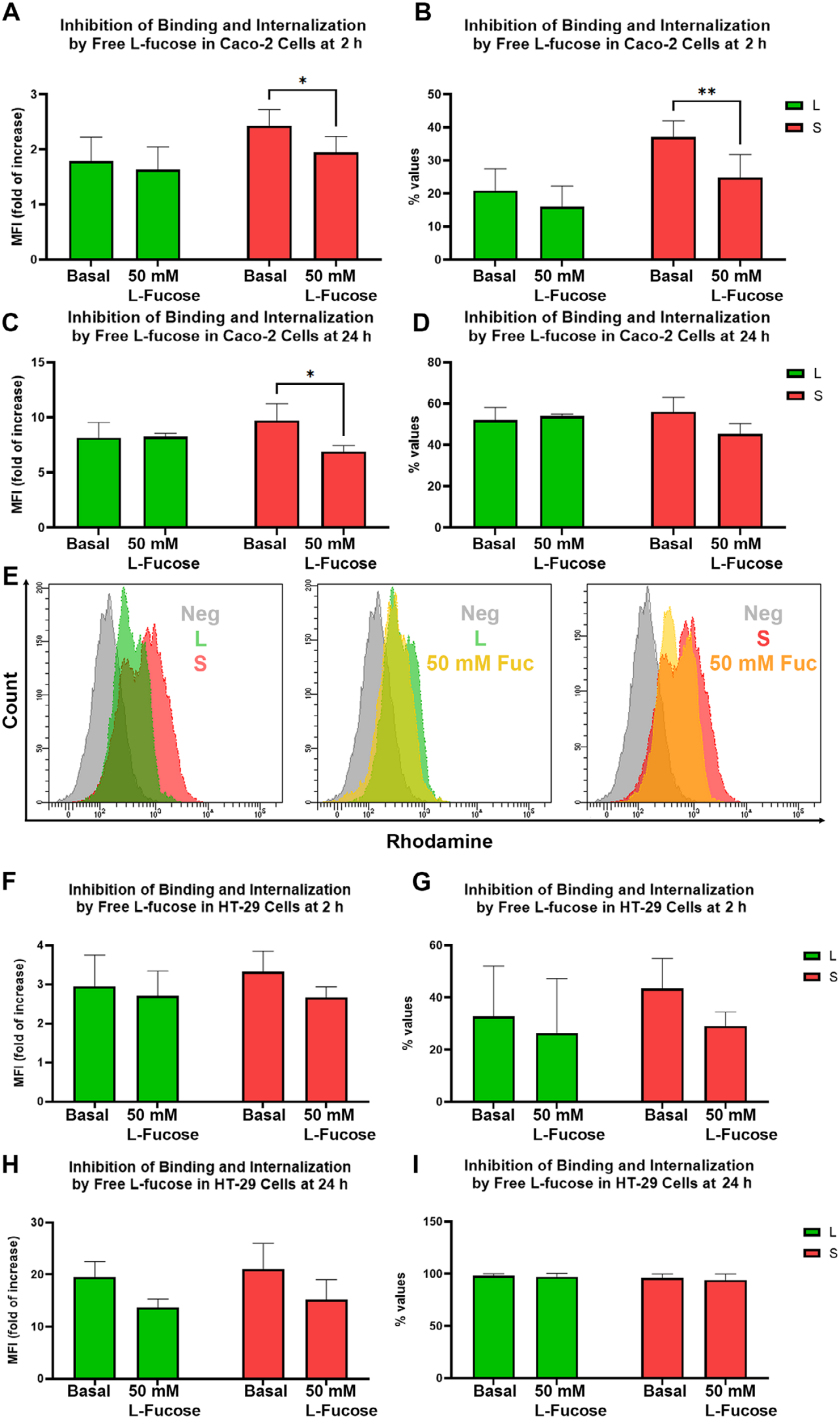
Competitive inhibition of L and S binding and internalization by free L‐fucose in intestinal cell lines. Quantitative analysis of the uptake of L (green) and S (red) was performed in Caco‐2 (A–E) and HT‐29 (F–I) cells at 2 and 24 h, in the presence or absence of 50 mM free L‐fucose. (A) MFI fold increase and (B) percentage values in Caco‐2 cells after 2 h of incubation. (C) MFI fold increase and (D) percentage values in Caco‐2 cells after 24 h. (E) Representative flow cytometry histograms for Caco‐2 cells at 2 h illustrating the uptake of L (green) and S (red) relative to negative control cells, and the subsequent reduction of uptake in samples treated with 50 mM L‐fucose (yellow). (F) MFI fold increase and (G) percentage values in HT‐29 cells after 2 h of incubation. (H) MFI fold increase and (I) percentage values in HT‐29 cells after 24 h.

To confirm whether the cellular uptake of fucosylated sweetosomes was mediated by specific ligand‐receptor recognition, a competitive inhibition study was performed using free L‐fucose as a competitor on Caco‐2 and HT‐29 cell lines. At the early incubation time point (2 h), the addition of an excess of free L‐fucose resulted in a moderate reduction in cellular binding, with significant inhibition levels ranging between 20% and 25% by both MFI and percentages analytic approach for Caco‐2 cells whereas the inhibition appeared modest and not significant in HT‐29 cells. Competition assays with free L‐fucose reveal that the preferential binding and internalization of sweetosomes are only partially mediated by the direct engagement of lectin receptors. The incomplete inhibition by the monovalent ligand L‐fucose, suggests that the sweetosome's cellular entry is driven by a multivalent ‘cluster effect’, which significantly enhances binding avidity compared to the free sugar [64] (Figure S5). Therefore, it is conceivable to attribute a great part of the superior internalization of sweetosomes to a multivalent “velcro effect”, which provides significantly higher binding avidity than non‐decorated blank liposomes, as highlighted by previous studies [65, 66]. This cooperative surface engagement ensures robust cellular adhesion. The differential inhibition of sweetosome uptake by free L‐fucose in HT‐29 versus Caco‐2 cells (Figure 4A–E vs Figure 4F–I) can be primarily attributed to their distinct phenotypic characteristics, specifically regarding mucin production and lectin expression profiles.

While Caco‐2 cells predominantly represent an absorptive enterocyte phenotype, HT‐29 cells are characterized by their ability to differentiate into mucus‐secreting goblet cells [67, 68, 69], able to create a competitive microenvironment trapping ligands suggesting a significant decrease of the competitive efficiency of exogenously added free L fucose compared to the multivalent avidity of sweetosomes, in agreement with several researchers [70]. Furthermore, the higher density of high‐affinity fucose‐binding lectins on the HT‐29 membrane leads to the “cluster glycoside effect” [71, 72] that free simple L‐fucose cannot effectively displace. In contrast, the absence of a significant mucus barrier in standard Caco‐2 cultures allows for a more direct interaction between sweetosomes and the apical membrane lectinic receptors [73], rendering the uptake process more susceptible to partial competitive inhibition (25%–30%) by free ligands. Consequently, the physiological differences in secretory activity and receptor accessibility explain why L‐fucose‐mediated inhibition appear modest and not significant in HT‐29 cells while being well detectable in the Caco‐2 model.

### 3.5 Sweetosome Internalization Begins With a Membrane Microdomain Caveolae‐Dependent Event, Which Better Defines Sweetosome Specificity and Affects the Features of Enclosing Endosomes

Next, the endocytic pathway of the two different vesicle‐based formulations were characterized. After their addition to a biological system, DDSs adhere to and enter the cells via distinct pathways. Various mechanisms of endocytosis can be involved in their internalization, including clathrin‐dependent, caveolae, and/or cholesterol‐rich lipid raft mechanisms. Particularly, lipid rafts are implicated in caveolae‐mediated endocytosis, a clathrin‐mediated pathway, and macropinocytosis. Rennick and coworkers reported Caco‐2 cells as not possessing caveolae because they lack CAV1 [74]; however, several researchers reported caveolae‐mediated entry of several viruses into Caco‐2 cells at the apical membrane, including recently the internalization and transport of SARS‐CoV‐2 [75]. Indeed, Bannunah and coworkers [46] describe in nonpolarized Caco‐2 cells (similar to those employed in this study), dynamin‐dependent endocytosis (including via clathrin‐ and caveolae‐dependent mechanisms), as the prevailing routes in the internalization of both positively and negatively charged nanoparticles. Very recently [76], Caco‐2 cells were further selected to validate the role of the caveolar pathway in colloidal nanoparticles/CNPs uptake.

**FIGURE 5:**
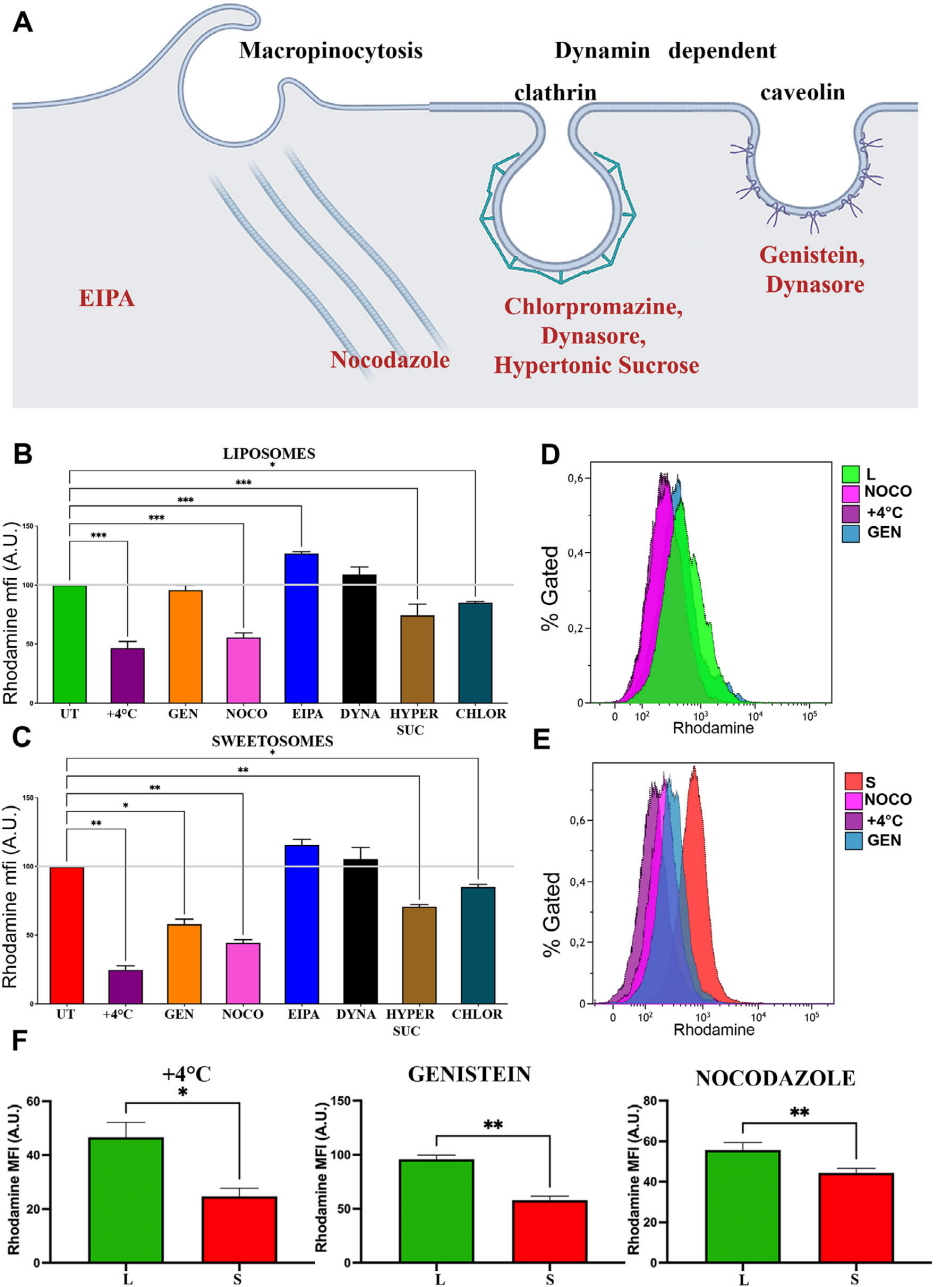
Blank liposomes and sweetosomes display differential responses to pharmacological inhibition with evidence of increased relevance for CvME. (A) Scheme of the impact of pharmacological inhibitors (in red) employed in this study on endocytic processes. L and S were used for uptake studies in the presence of endocytosis inhibitors. (B,C) L and S were utilized for uptake studies alongside endocytosis inhibitors targeting various pathways, specifically for clathrin‐mediated endocytosis (chlorpromazine hydrochloride, dynasore, and hypertonic sucrose), caveolae‐mediated endocytosis (genistein), macropinocytosis (EIPA) micropinocytosis (nocodazole), and ATP‐dependent processes (+4°C) as detailed in Table 2 of the Materials and Methods section. MFI measures the percentage of L and S uptake, determined through flow cytometry, and normalized with rhodamine MFI values in the untreated condition (UT) at 37°C (UT corresponds to 100% uptake). (D,E) Representative cytometric overlay histogram of L and S uptake at different inhibitor conditions (NOCO, +4°C, GEN). (F) Comparison of specific inhibitors (+4°C, GEN, NOCO) effects on L and S uptake.

Several inhibitors were employed to investigate these processes (i.e., endocytosis via specific receptors in clathrin‐mediated [CME]), caveolae‐mediated [CvME], and clathrin‐ and caveolae‐independent endocytic pathways) (Figure 5A) [77].

Flow cytometry quantification of rhodamine fluorescence was employed to study blank liposome and sweetosome uptake in Caco‐2 cells. After 2 h of exposure, the addition of inhibitors—EIPA, dynasore, hypertonic sucrose, and chlorpromazine—resulted in a comparable level of inhibition of the uptake. MFI data were adjusted to reflect the overall uptake of liposomes at 37°C under control conditions (without inhibitors), establishing a 100% reference point for comparison (Figures 5B and 4C) [78, 79].

Low temperature (4°C), or treatment with genistein or nocodazole (CvME inhibitors) resulted in a stronger inhibition with sweetosomes than blank liposomes (Figure 5D,E). These findings highlight a striking difference in the uptake mechanisms between blank liposomes and sweetosomes. Although CME and CvME processes coexist for the internalization of both formulations, Figure 5D shows that CvME was prevalent in sweetosome uptake (Figure 5F). This upstream feature appears to condition the features of the downstream endosomes, which, normally, during endosomal maturation, change the endosomal environment from neutral to slightly acidic (pH ∼ 6.5 in early endosomes, pH ∼ 5.5 in late endosomes, and pH ∼ 4.7 in lysosomes) [80].

Scientific literature since 2009 [81] presents conflicting views on caveolar trafficking, describing it as either a path to lysosomal degradation or a privileged route that bypasses it. Recently [82], liposomal nanoparticles were shown to induce CvME—via STX6 [syntaxin] upregulation—potentially avoiding lysosomal sequestration and boosting siRNA delivery. Since cellular uptake and endosomal escape remain the critical bottlenecks for nanomedicines [83, 84] to prevent enzymatic breakdown [80, 85], the endolysosomal system of these colon adenocarcinoma cells was thoroughly investigated for both formulations.

### 3.6 Sweetosomes Exhibit Longer Persistence, With Intracellular Scattering Through the Endosomal System: Mapping Endosomal Escape Independently by the Specific Payload

The release of the payload prior to complete lysosomal maturation is a crucial stage for efficient delivery, known as endosomal escape [86]. Although the formulations used in this experiment did not contain specific payloads, the endolysosomal involvement in their intracellular behavior was evaluated. This kind of study may support the choice of the most useful combination of a drug with a specific nanovector.

Particularly, we investigated the acidity level of late endosomes, characterized by a progressively low pH [87]. For this purpose, we combined LysoSensor and LysoTracker Deep Red (LTDR) in cells treated with the two different liposomal formulations.

**FIGURE 6:**
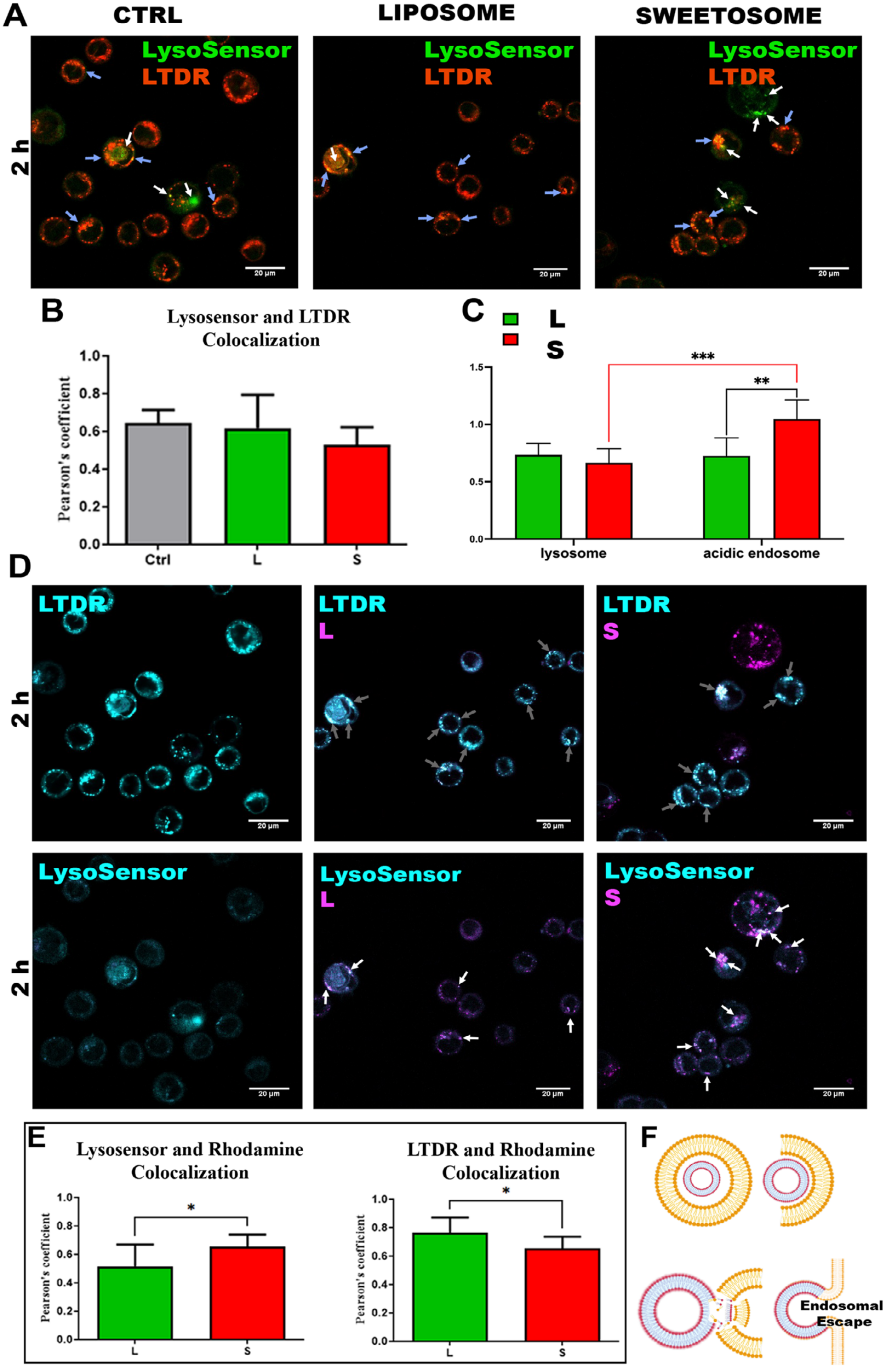
An investigation of organelle acidity points out specific modulation by sweetosomes, suggesting a certain benefit in the process of endosomal escape: (A) Single confocal optical sections of Caco‐2 cells from different treatments (Ctrl, liposomes, sweetosomes) at 2 h: in green LysoSensor, in red LTDR. (B) Statistical histogram of LysoSensor and LTDR colocalization using Pearson's coefficient. (C) The ratio of mean fluorescence of lysosomes and acidic endosomes in liposome (green)‐ and sweetosome (red)‐treated cells. (D) Single confocal optical sections of Caco‐2 cells display the colocalization of liposomes and sweetosomes (magenta) with LTDR (cyan, above, indicated by gray arrows) and LysoSensor (cyan, below, indicated by white arrows) at 2 h. Arrows indicate the areas of colocalization. (E) Statistical histograms of LysoSensor (left) and LTDR (right) colocalization with rhodamine, labeled L and S, using Pearson's coefficient. (F) Scheme of endosomal escape induced by liposomes.

LysoSensor is a probe useful to monitor pH within lysosomes or any other compartment (e.g., endosomes) [88]. In contrast, the LTDR dye only marks typical acidic organelles, such as lysosomes [89]. LysoSensor has the additional advantage of tracking fluctuations in pH. Co‐labelling with both probes can give information on endosome acidification of endosome‐lysosome fusion in the presence of the specific fluorescent nanocarrier. The co‐labelling is safe for the cells and is possible from a methodological point of view as we performed previous tests on control cells. All evaluations were performed in short times (15–20 min); therefore, the organelle alkalinization induced by LysoTracker dyes if the dyes’ permanence is prolonged [90] was avoided. Our newly optimized method allowed us to depict the different colocalization of LTDR and green acidic endosomes (LysoSensor Green) in Caco‐2 cells in control samples treated with blank liposomes (L) and sweetosomes (S) (Figure 6). Blue arrows indicate the colocalization of both dyes (considered to mark lysosomes; Figure 6A), and white arrows show only green punctate fluorescence (acidic endosomes). The Pearson coefficient of colocalization is reported in Figure 6B, whereas, in Figure 6C, the ratio “treatment:control” is shown from the quantification of LTDR MFI (referring to the whole acidic compartment, mainly lysosomes) and the correlation between LysoSensor and LTDR ratios. This calculation enables quantifying the two different compartments by FC and corresponds to the confocal analyses, showing that sweetosomes induced an increase in acidic endosomes but not in lysosomes.

Merged confocal microscopy images of Caco‐2 cells treated with rhodamine‐labeled liposomes and sweetosomes for colocalization with LTDR and LysoSensor (Figure 6D) demonstrated that sweetosomes exhibited a clear colocalization with acidic endosomes (indicated by white arrows). This feature was notably absent for blank liposomes in the investigated intestinal carcinoma cell line (Figure 6E), representing a condition potentially applicable to project‐specific combinations of different drugs and delivery nanosystems. Furthermore, these aspects should be monitored across different cell types, as they can differ significantly in their endolysosomal behavior; accordingly, the liposome formulations show a different endosomal escape efficiency (Figure 6F) based on the specific cell type. Indeed, to better characterize the features of these endosomal vesicles—which primarily derive from caveolae‐mediated endocytosis—the labeling protocol was implemented by adding anti‐CD81, anti‐CD63 (Tetraspanin 30 /LAMP3), and anti‐LAMP1/CD107a mAbs. In fact, LAMP3 and LAMP1 (together with EEA1) represent widely used markers for early and late endosomal compartments with a multivesicular appearance [91].

**FIGURE 7:**
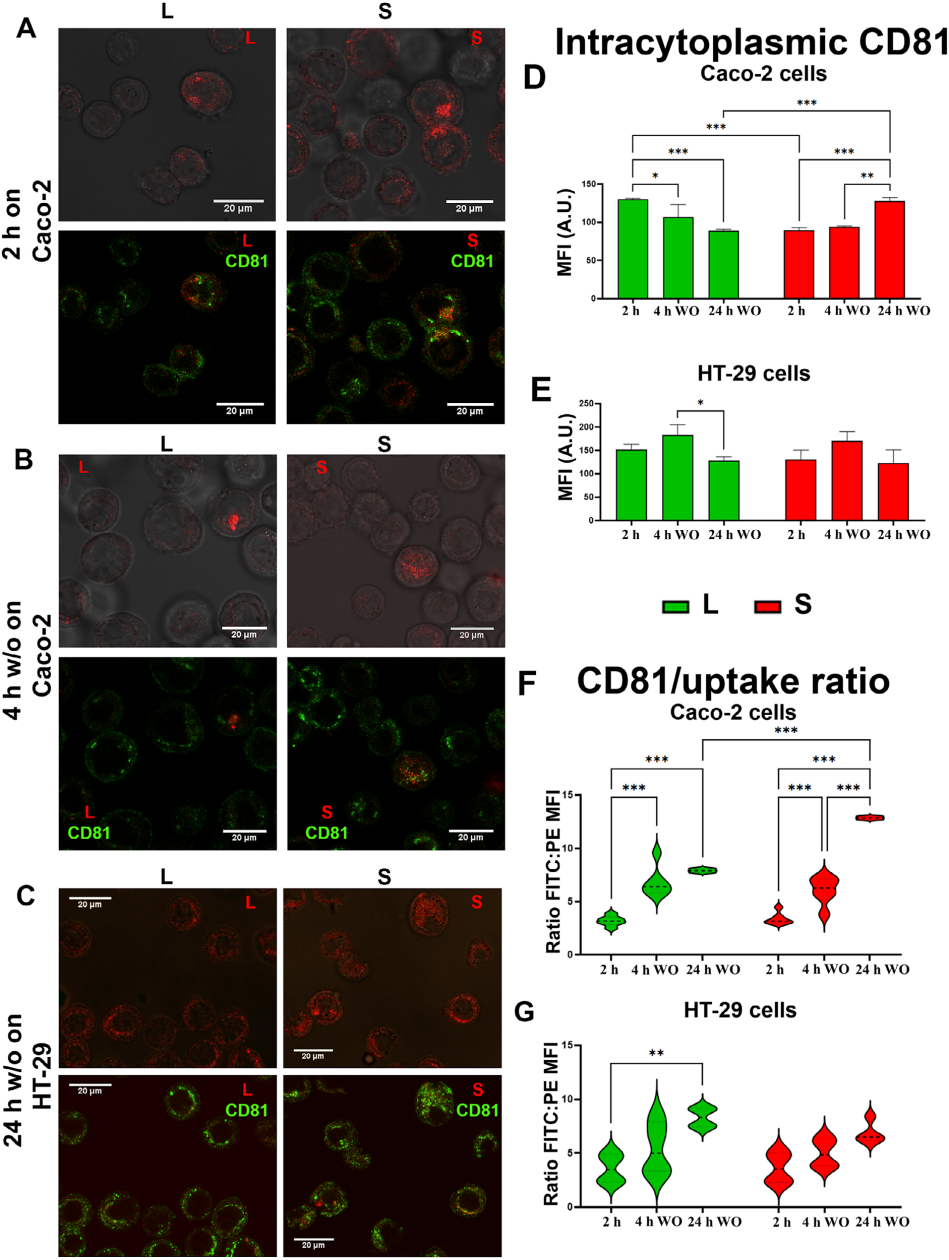
Blank liposomes and sweetosomes enter the cell and traffic in different endosomal vacuoles. (A–C) Single confocal optical sections of CD81 distribution (green) during pulse and chase experiments of liposomes (red) and sweetosomes (red) in both cell lines, after 2 h of pulse and 4 h of chase in Caco‐2 cells (A, B) and 24 h of chase in HT‐29 cells (C). In green CD81, in red liposomes and sweetosomes. (D,E) Statistical histograms of intracytoplasmic CD81 expression after 2 h of pulse and 4 and 24 h of chase of L (green) and S (red) in Caco‐2 and HT‐29 cells. (F,G) Violin plots and statistical analysis of the ratio between intracytoplasmic CD81 and L, S uptake after 2 h of pulse and 4 and 24 h of chase in Caco‐2 and HT‐29 cells.

CD81 is a highly conserved four‐transmembrane protein in mammals and is widely expressed in many tissues. It belongs to the tetraspanin family, and these proteins have a role in the regulation of many biological processes such as cell‐cell adhesion, fusion, signal transduction, proliferation and differentiation [92] and regulate endosomal network dynamics [93]. CD63 and CD81 are implicated in the cargo sorting process to intralumenal vesicles/ILVs during the inward budding process, forming multi‐vesicular bodies/MVBs. Indeed, LAMP1/CD107a marks late endosomes (LEs), demonstrating a process of inward budding. Considering this, the quantification of CD81 expression and the evaluation of its distribution after the uptake of the two different formulations (in both cell lines, after 2 h of pulse, followed by 4 and 24 h of chase, Figure 7), not only shows cell and time‐dependent behavior but also a specific trend for blank liposomes and sweetosomes. Figure 6A–C highlights intracellular CD81 and blank liposomes and sweetosomes internalized by both cell lines. The decrease in intracellular CD81 after blank liposome endocytosis seems to indicate that CD81 is being trafficked to lysosomes for degradation (Figure 6D,E), rather than being recycled back to the cell surface or incorporated into exosomes, a behavior unobserved for sweetosome‐treated cells, particularly evident in HT‐29 confocal images, after 24 h (Figure 6C).

Furthermore, calculated MFI ratios between CD81values/uptake values (Figure 6F,G) indicate a relevant increase after 4 h and 24 h, particularly for sweetosomes, significantly peaking with respect to blank liposomes in Caco‐2 cells. These findings parallel the greater intracellular persistence of sweetosomes: CD81 levels move in agreement with a different involvement of intralumenal vesicles within multi‐vesicular bodies, because they can arise from the endosomal network and the membrane recycling apparatus [93]. However, HT‐29 cells show a different trend for this ratio, and particularly for sweetosomes, not revealing any relevant or significant difference.

To better address this point, CD63 was also considered for its distribution in cells, when rhodamine‐stained liposomes were analyzed in the medium, revealing liposome exits, undoubtedly derived from releasing processes. CD63 differs from CD81: the former is mainly intracytoplasmic, whereas the latter is present at both the plasma membrane and with intracellular localization. A key role of CD63 in regulating the interactions between endosomal and autophagy processes (limiting cellular signaling activity) was also pointed out by Hurwitz and coworkers [94] in both non‐infected and virally infected cells. Indeed, recently, a subclass of non‐proteolytic endosomes was identified at the pre‐lysosomal stage as the compartment of origin of CD63‐positive (CD63^+^) exosomes [91].

**FIGURE 8:**
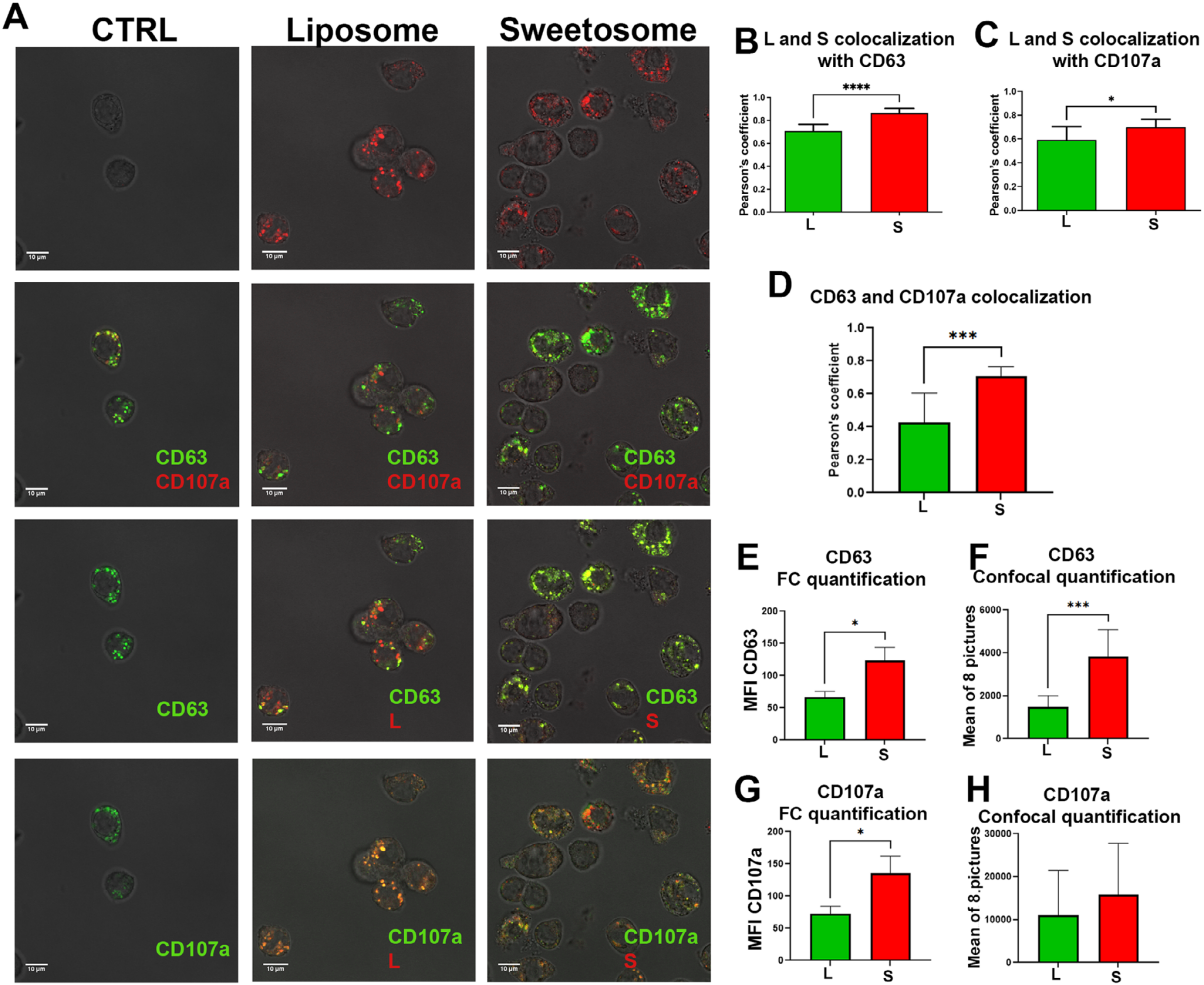
Sweetosomes with their fucose residues act on maturation and endosome functions. (A) Single confocal optical sections of HT‐29 cells treated (Ctrl, liposome, and sweetosome) at 72 h of continuous uptake: The first line shows the rhodamine fluorescence (red) of L and S. The second line shows CD63 (green) and LAMP1/CD107a (red). The third line shows CD63 (green) and rhodamine fluorescence (L or S in red). The fourth line shows LAMP1/CD107a (green) and rhodamine fluorescence (L or S in red). (B) Statistical histogram of CD63 and L, S colocalization using Pearson's coefficient. (C) Statistical histogram of LAMP1/CD107a and L, S colocalization using Pearson's coefficient. (D) Statistical histogram of CD63 and LAMP1/CD107a colocalization in liposomes and sweetosomes using Pearson's coefficient. (E,F) Statistical histograms of CD63 fluorescence in L and S using FC and confocal quantification by calculating eight pictures as mean values. (G, H) Statistical histogram of LAMP1/CD107a fluorescence in L and S using FC and confocal quantification by calculating the mean of eight pictures.

Therefore, data on intracytoplasmic distribution of CD63^+^ vacuoles/vesicles, also expressing LAMP1/CD107a for both blank liposome and sweetosome uptake, are shown in Figure 8A. These findings depict the endolysosomal involvement of the condition mimicking an in vivo administration of liposomes for enhanced bioavailability of water‐insoluble drugs and are related after 72 h continuous uptake. The drug finally involved in the current study is curcumin, which is finally encapsulated in the two formulations (see the following paragraph). CD63 and LAMP1/CD107a patterns highlight a significantly higher colocalization of the two molecules in sweetosome‐treated cells (Figure 8B,C): it is possible to assign to these structures the same features identified by Verweij and coworkers, with evidence of LAMP1/CD107a expression (late endosomes) in CD63^+^ endosomes (Figure 8D), functional to liposome rupture/degradation and to cargo delivery, in agreement with Ahmal and coworkers, who found that the majority of the liposome payload (siRNAs) was identified in late endosomes after 2 h, whereas siRNAs were observed in the cytosol after 4 h, demonstrating their escape from endosomes [95]. The subsequent evaluation of blank liposomes and sweetosomes colocalization in these endosomes points out opposite MFI values for LAMP3 and LAMP1: in fact, CD63 in sweetosomes and LAMP1/CD107a in liposomes, suggested that blank liposomes reach mainly lysosomes, containing a plethora of enzymes that can rapidly destroy various types of particles (Figure 8E–H) [95]. Such analyses help to uncover how rapidly lipid‐based nanoformulations, or other delivery systems deteriorate, aiming to preserve them from immediate lysosomal degradation and to design an acceptable delivery vector for cells that can evade endosomes and access the entire cytoplasm [95].

### 3.7 Fucosylated Sweetosomes Ensure a More Efficient Targeting of Intestinal Cancer Cells Compared to Liposomes and a Heading Toward Different Acidic Organelles, Enhancing the Drug's Effectiveness

Because endocytosis is the typical pathway for liposome internalization, endocytosed liposomes must also be able to break out of the endosomal vesicles during the endocytic process or to fuse them with endosome membranes to deliver their cargo to cytosolic compartments efficiently [96, 97].

After cell internalization, blank liposomes and sweetosomes are sequestered in endosomal acidic compartments but with different characteristics. As demonstrated in the previous paragraphs, starting at 2 h, they involve endosomal vesicles with different pH, and during time, are also naturally able to modify the pH within endosomes, thereby interfering with complete lysosome maturation. These data identify LEs as desirable gateways for cell penetration. Such findings fit with Erazo‐Oliveras and coworkers, highlighting that LEs and the phospholipid bis(monoacylglycero)phosphate when particularly enriched, can be exploited for the development of future delivery agents [98].

**FIGURE 9:**
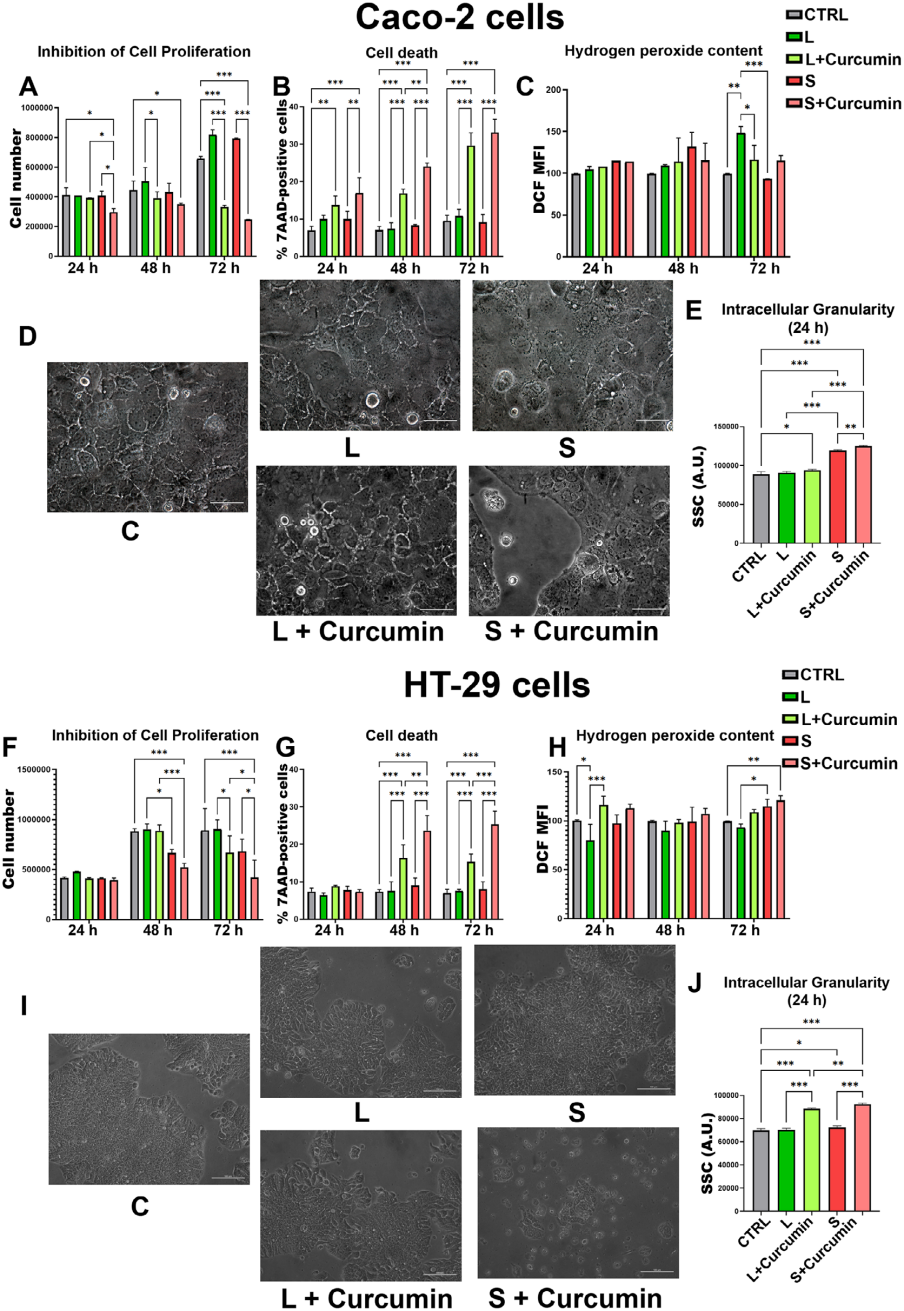
Sweetosomes prevail in the tug‐of‐war for curcumin effectiveness in both Caco‐2 and HT‐29 cells. (A) Statistical histogram of absolute counts of Caco‐2 expressed in cell number, (B) cell death as a percentage of 7‐AAD‐positive cells, and (C) hydrogen peroxide content in DCF MFI. These values are presented under different conditions (CTRL, L, L+curcumin, S, S+curcumin) from 24 h to 72 h. (D) Images of brightfield microscopy of Caco‐2 treated cells after 24 h. (E) Statistical histogram of intracellular granularity in Caco‐2 cells at 24 h was observed through the SSC parameter. (F) Statistical histogram of absolute count of HT‐29 expressed in cell number, (G) cell death as percentage of 7‐AAD‐positive cells, and (H) hydrogen peroxide content in DCF MFI. These values are presented under different conditions (CTRL, L, L+curcumin, S, S+curcumin) from 24 to 72 h. (I) Images of brightfield microscopy of HT‐29 treated cells after 72‐h incubation. (J) Statistical histogram of intracellular granularity in HT‐29 cells at 24 h, observed through the SSC parameter.

The study of the different endo/lysosomal system involvement has its high‐water mark in evaluating sweetosome performance through the effects of curcumin, as model drug, after its encapsulation, uptake and intracellular release. Curcumin is a natural polyphenol extracted from turmeric. Numerous studies have demonstrated that curcumin is an effective anticancer drug that modifies different intracellular signaling pathways [99]. Curcumin's therapeutic utility is severely constrained by its short half‐life in vivo, low water solubility, poor stability, quick metabolism, low oral bioavailability, and potential gastrointestinal discomfort with high oral doses [100]. One of the most practical solutions to these issues is the development of targeted DDS‐based nanomaterials [56]. Effects of the exposure to curcumin‐loaded and blank liposomes and sweetosomes were evaluated after 24, 48 and 72 h, and data from 3 independent experiments are reported in Figure 9A–E for Caco‐2 and 9 F‐J for HT‐29 cancer cell lines (Figure S6). Cell counts (to monitor proliferation), cell death induction, and ROS modulation were evaluated along with ancillary light microscopy images (Figure 9D,I), confirming FC quantification. Based on all parameters investigated, curcumin was more effective if contained and released by sweetosomes, even though inside each formulation the same drug concentration was charged.

Finally, the evaluation of cell granularity (Figure 9E,J), from FC side scatter values, confirms a higher “diffusion” of sweetosomes in the cytoplasm, particularly evident for Caco‐2 cells at 24 h after their administration, when cells still do not divide and therefore their dilution in daughter cells does not have an impact. Indeed, the increase in granularity was also evident in confocal microscopy images.

### 3.8 Internalization of Blank Liposomes and Sweetosomes in Immune Cells: Ex Vivo Assessment in Whole Peripheral Blood Highlights Their Differential Internalization and Striking Translational Aspects

**FIGURE 10:**
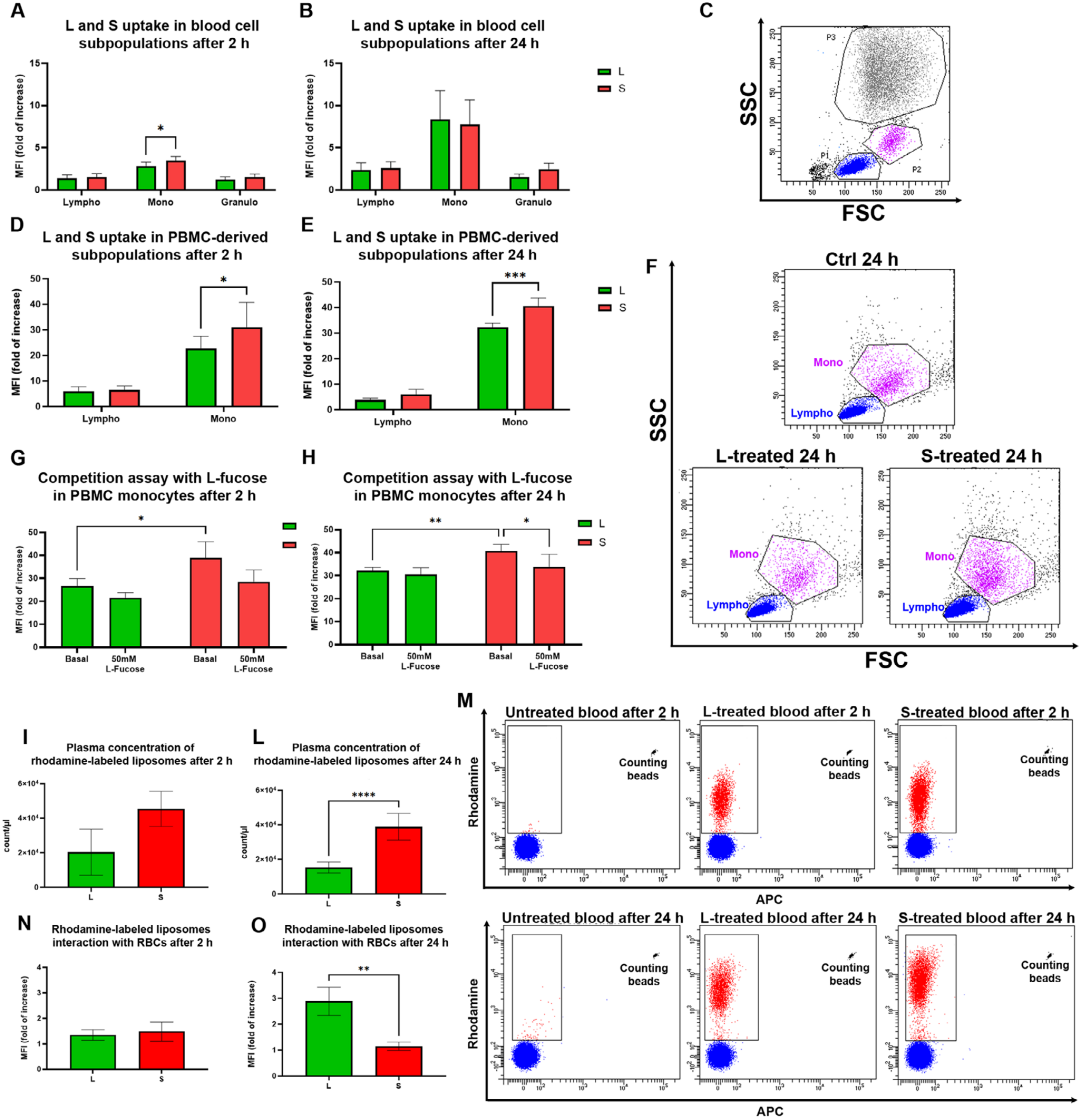
Cellular uptake and competitive inhibition of L and S formulations in blood cell subpopulations and their comparative interaction with plasma and erythrocytes suggest striking translational aspects. (A–H) Quantitative analysis of L (green) and S (red) internalization in primary blood cells and PBMC‐derived subpopulations. (A) MFI (fold of increase) in blood cell subpopulations (lymphocytes, monocytes, and granulocytes) after 2 and (B) 24 h of incubation. (C) Analysis of uptake in PBMC‐derived lymphocytes and monocytes after 2 and (D) 24 h. (E) Representative flow cytometry dot plots (FSC vs. SSC) illustrating the gating strategy for lymphocytes (blue) and monocytes (purple) in control, L‐treated, and S‐treated samples at 24 h. (F) Competition assay in PBMC‐derived monocytes at 2 and (G) 24 h, comparing basal uptake with samples treated with 50 mM L‐fucose. (I–O) Plasma permanency and aspecific adhesion on RBCs. (F) Plasma concentration (count/µL) of rhodamine‐labeled liposomes at 2 and (B) 24 h post‐treatment, showing a significantly higher persistence of the S formulation at the later time point. (C) Representative flow cytometry dot plots (APC vs. Rhodamine) of untreated, L‐treated, and S‐treated blood samples at 2 (top) and 24 h (bottom). Blue: plasma components; Red: L or S rhodamine labelled liposomes; Black: counting beads. (D) Quantitative analysis of liposomal interaction with Red Blood Cells (RBCs), expressed as MFI (fold of increase) at 2 and (E) 24 h, highlighting a differential association pattern between L and S formulations over time. Six different experiments (from six different donors) were performed in duplicate.

The experimental data indicate that sweetosomes exhibit a significant, albeit moderate (∼25%), increase in internalization by circulating monocytes compared to non‐decorated counterparts within the first 2 h of incubation (Figure 10A) whereas, other leukocyte subpopulations—specifically lymphocytes and granulocytes—exhibit minimal uptake, with no statistically significant differences between the two formulations. Furthermore, in healthy donors, a distinct and rare population of CD123+/HLA‐DR^low monocytes was identified within the monocytic morphological gate, with the goal of collecting cells with high CD206 and CD209 expression [101]. In this static WhB system, the major uptake (∼25%) of sweetosomes suggests a critical kinetic window of active targeting. During this 2 h initial phase, the fucose ligands appear biologically accessible to monocytic C‐type lectin receptors (Figure 10A) and to putative circulating monocyte‐derived dendritic cells (Figure S7), suggesting a liposomal surface preceding the full maturation of the protein corona [102, 103]. As incubation progresses toward 24 h, the observed equilibrium in uptake degree (Figure 10B) demonstrates the vanishing of biological distinction between sweetosomes and blank liposomes. The comparison between ex vivo whole blood data and data from gradient‐separated peripheral blood mononuclear cells (PBMCs) (Figure 10C,D) indicates that whole blood sweetosome surface is likely covered by a dense “hard corona” of adsorbed plasma proteins, as extensively discussed by Mojarad‐Jabali et al., [104] masking during time the fucose residues that, in RPMI, achieve optimal accessibility at both 2 and 24h. These findings are in strong agreement with current literature [105, 106], highlighting some further advantages for their tissue‐directed intravenous administration [107]. Therefore sweetosomes appear to maintain high bioavailability, ensuring they remain available for successful delivery to peripheral tissues and highlighting that interplay of fucose moieties with the plasma corona reach a more favorable biologic identity, at least in the early time course, as focused by several authors [108, 109].

To further analyze the interaction of liposomes with the physiological environment, the plasma‐residing time of both formulations and their potential non‐specific adhesion to red blood cells (RBCs) were evaluated. A significantly higher recovery of sweetosomes was found in the plasma fraction at 2 h; this trend becomes even more pronounced and statistically significant after 24h (Figure 10E,F). Contemporary, rhodamine fluorescence evaluated on RBCs demonstrated values significantly lower in sweetosome‐treated whole blood than blank liposomes. In the current blood static model, this prolonged plasma persistence of sweetosomes can also be attributable to the minimized non‐specific binding toward RBCs, which can effectively prevent their premature clearance from the bloodstream, in vivo [110, 111].

## 4 Conclusion

Sweetosomes, produced through a simple one‑step microfluidic process, emerge as a highly promising platform for targeted drug delivery thanks to their intrinsic stealth behavior, reduced interaction with blood components, and prolonged circulation. Their fucosylated surface supports multivalent recognition by lectinic receptors expressed on colorectal cancer cells, enabling more selective uptake and sustained intracellular retention. The system also shows favorable trafficking features, with a tendency to localize in late endosomal compartments, an advantageous site for the release of biological payloads, while the proposed workflow for quantifying exocytosis further strengthens their evaluation as delivery vectors. Building on these properties, PEGylation represents a logical next step to further extend systemic permanence and maximize tissue‑targeting efficiency.

Overall, the study positions fucosylated sweetosomes as a robust and versatile nanocarrier platform, combining selective cell engagement, intracellular persistence, and promising pharmacokinetic behavior.

## Author Contributions

The manuscript was written through the contributions of all authors. All authors have given approval to the final version of the manuscript.

## Funding Information

This work has been published by the European Union—NextGenerationEU—under the Italian Ministry of University and Research (MUR) National Innovation Ecosystem grant ECS00000041—VITALITY—CUPH33C22000430006.

## Conflicts of Interest

The authors declare no conflicts of interest.

## Supporting information




**Supporting File**: adhm71254‐sup‐0001‐SuppMat.pdf.

## Data Availability

The data that support the findings of this study are available from the corresponding author upon reasonable request.
