# Supplementary material for: One‐Step Microfluidic Manufactured Fucose‐Decorated Sweetosomes Choose the Time and the Road for Their Intracellular Journey to Cancer Treatment
Source: Adv Healthc Mater. 2026 May 13;15(24):e05617. doi: 10.1002/adhm.202505617 (PMC13307633; doi:10.1002/adhm.202505617)
Supplement: Supplementary file 1 — Supporting File: adhm71254‐sup‐0001‐SuppMat.pdf. [file ADHM-15-0-s001.pdf]

SUPPLEMENTARY MATERIALS

One-step microfluidic manufactured fucose-decorated sweetosomes choose the time and the road for their intracellular journey to cancer treatment

Mattia Tiboni [1], Mariele Montanari [1], Shiva Khorshid [1], Michele Verboni [1], Andrea Duranti[1], Simone Lucarini [1], Daniele Lopez [1], Annalisa Aluigi [1], Gianluca Morganti [1], Michele Menotta [1], Giovanna Panza [1], Daniel J. Klionsky[3], Barbara Canonico [1] & Luca Casettari [1]*

[1] Department of Biomolecular Sciences (DISB), University of Urbino Carlo Bo, 61029 Urbino, Italy
[2] Institute for Experimental Molecular Imaging (ExMI), RWTH Aachen University Hospital, Aachen, Germany
[3] Life Sciences Institute, University of Michigan, Ann Arbor, MI, 48109, USA

** Corresponding author: barbara.canonico@uniurb.it*

Contributing authors: mattia.tiboni@uniurb,it, mariele.montanari@uniurb.it, s.khorshid@campus.uniurb.it, michele.verboni@uniurb.it, andrea.duranti@uniurb.it, simone.lucarini@uniurb.it, daniele.lopez@uniurb.it, annalisi.aluigi@uniurb.it, gianluca.morganti@uniurb.it, michele.menotta@uniurb.it, giovanna.panza@uniurb.it, klionsky@umich.edu, luca.casettari@uniurb.it

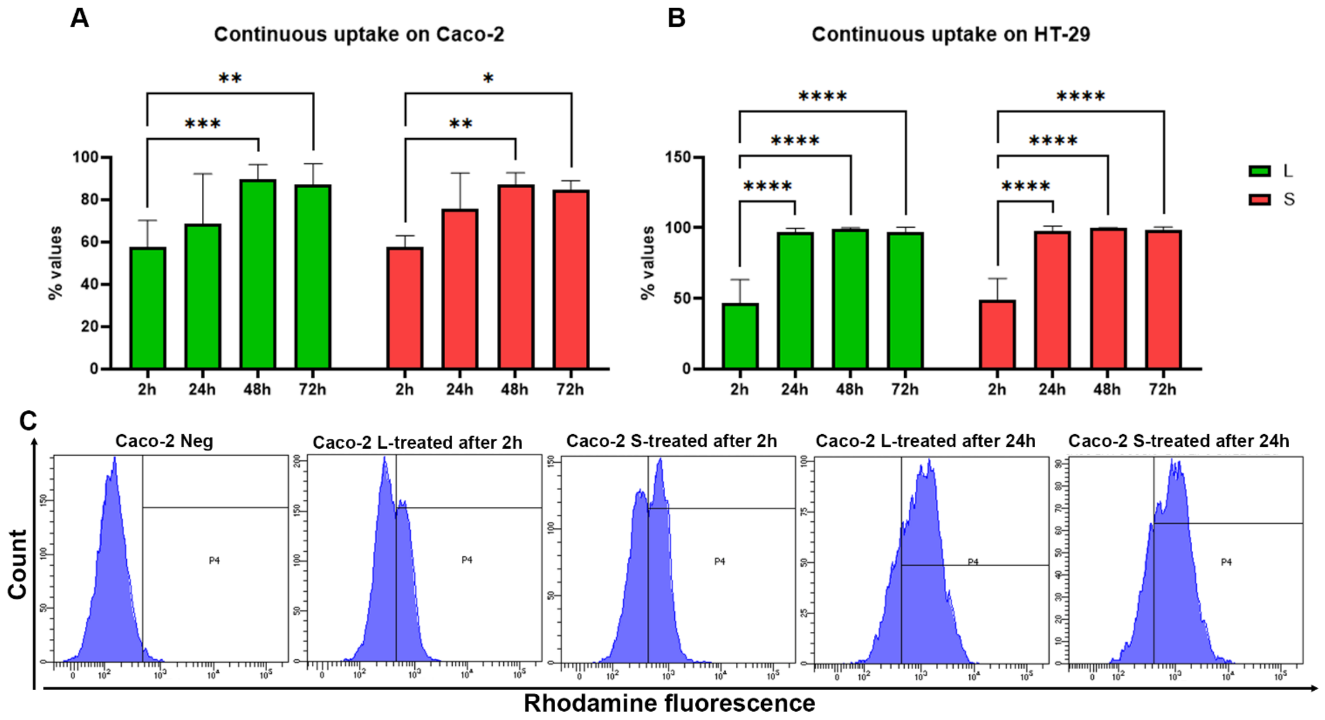

Supplementary Figure 1: Continuous cellular uptake of L and S formulations in intestinal epithelial models. (A–B) Quantitative analysis of the percentage uptake of Rhodamine-labeled L (green bars) and S (red bars) treatments in Caco-2 (A) and HT-29 (B) cell lines over a time course of 2, 24, 48, and 72 hours. Both cell lines show a time-dependent increase in internalization, reaching a plateau after 24/48 hours. (C) Representative flow cytometry histograms illustrating the shift in Rhodamine fluorescence intensity in Caco-2 cells. The panels show untreated cells (Neg) compared to cells treated with L and S formulations after 2h and 24h of incubation. The P4 gate indicates the population of cells positive for uptake.

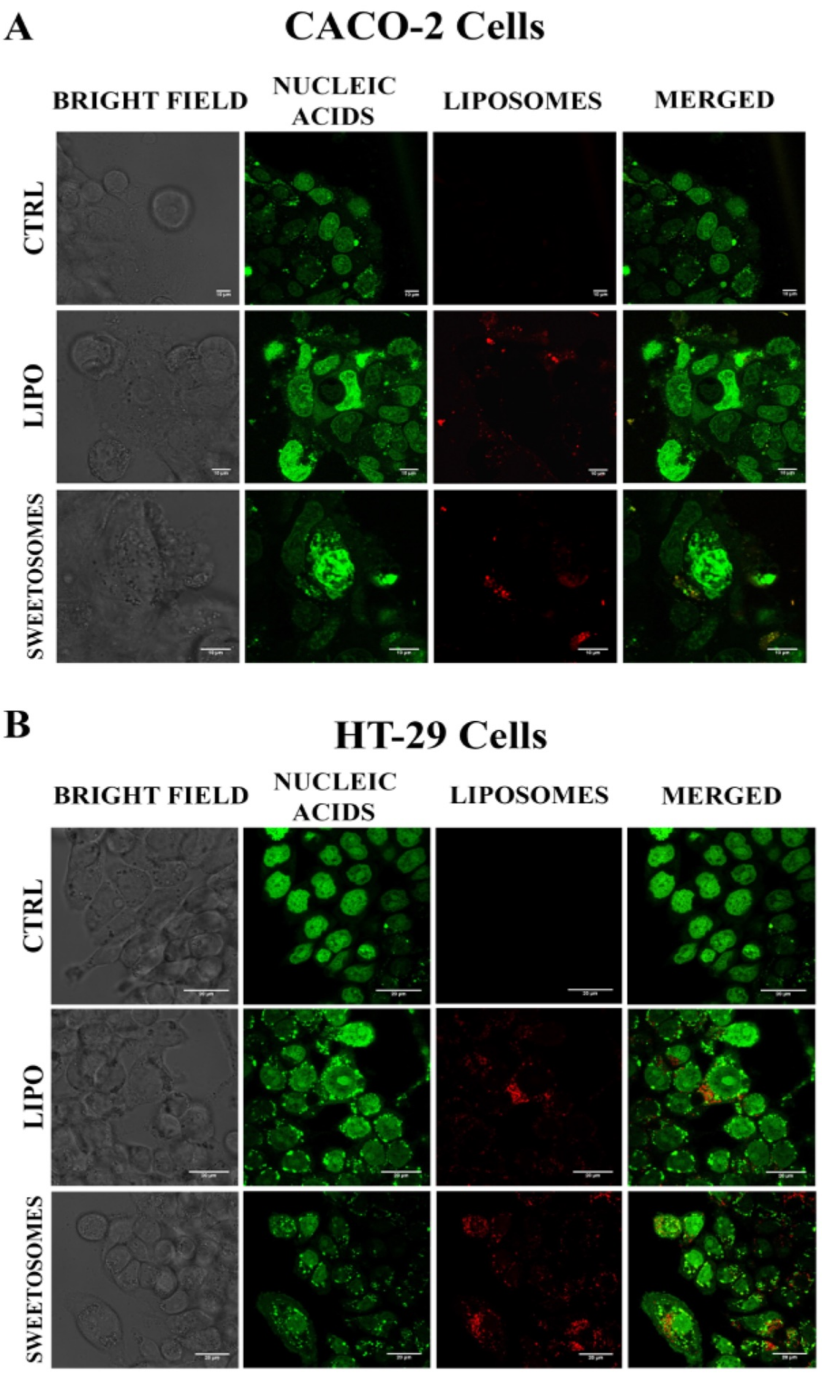

Supplementary Figure 2: Intracellular localization of the two liposome formulations after 2 h of continuous uptake. (A) Single confocal optical sections of nucleic acids (green) liposomes (red) and sweetosomes (red) in Caco-2 cells: CTRL, liposome, and sweetosome. (B) Single confocal optical sections of nucleic acids (green) and liposomes (red) and sweetosomes (red) in HT-29 cells: CTRL, liposome, and sweetosome.

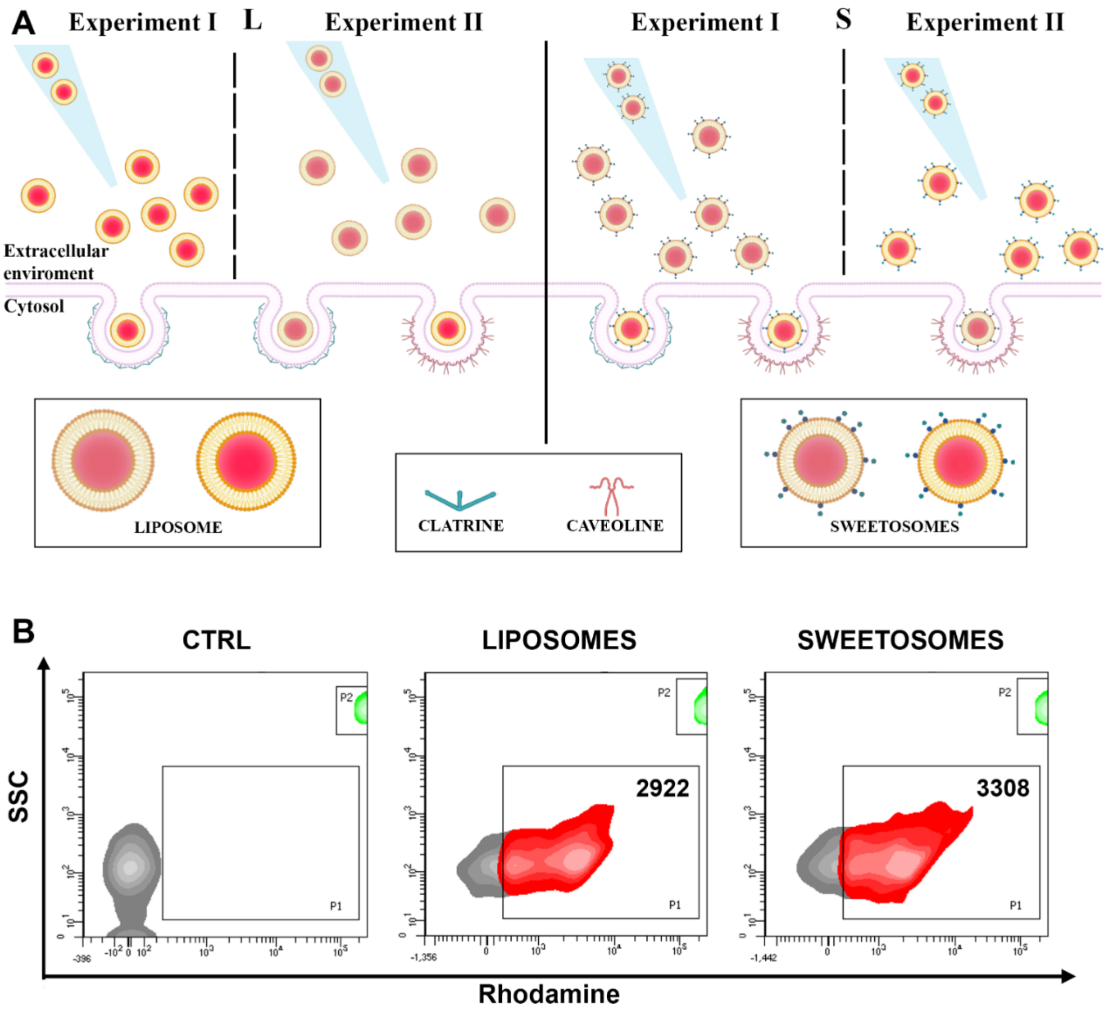

Supplementary Figure 3: Experimental setup to standardize the amount of liposome fluorescence in uptake experiments. (A) The experimental setting scheme, providing the fluorescence intensities of each formulation, can correctly evaluate the uptake degree for each batch of liposomes and sweetosomes tested in the different experiments. (B) The gating strategy used to analyze rhodamine fluorescence of liposomes and sweetosomes (in red) in mediums of different conditions (Ctrl, liposomes and sweetosomes). In the CTRL there is an absence of rhodamine fluorescence, in liposomes it is present with 2.922 MFI, and in sweetosomes with 3.308 MFI. Beads are represented in gate P2.

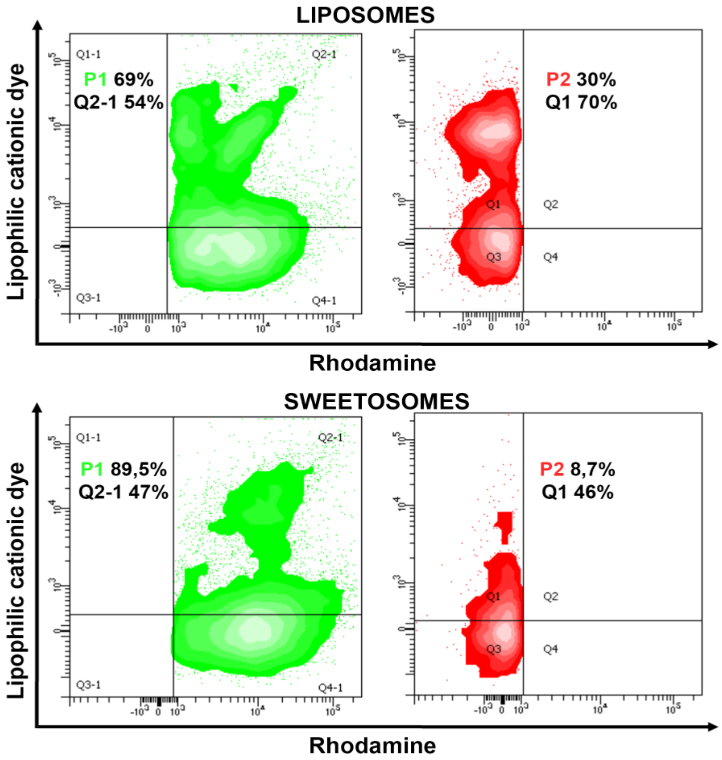

Supplementary Figure 4: Density plot represents the evaluation of formulation (L and S) stability after 30 days, using cationic lipophilic fluorescent probes that fluoresce only when label undamaged nanovesicles.

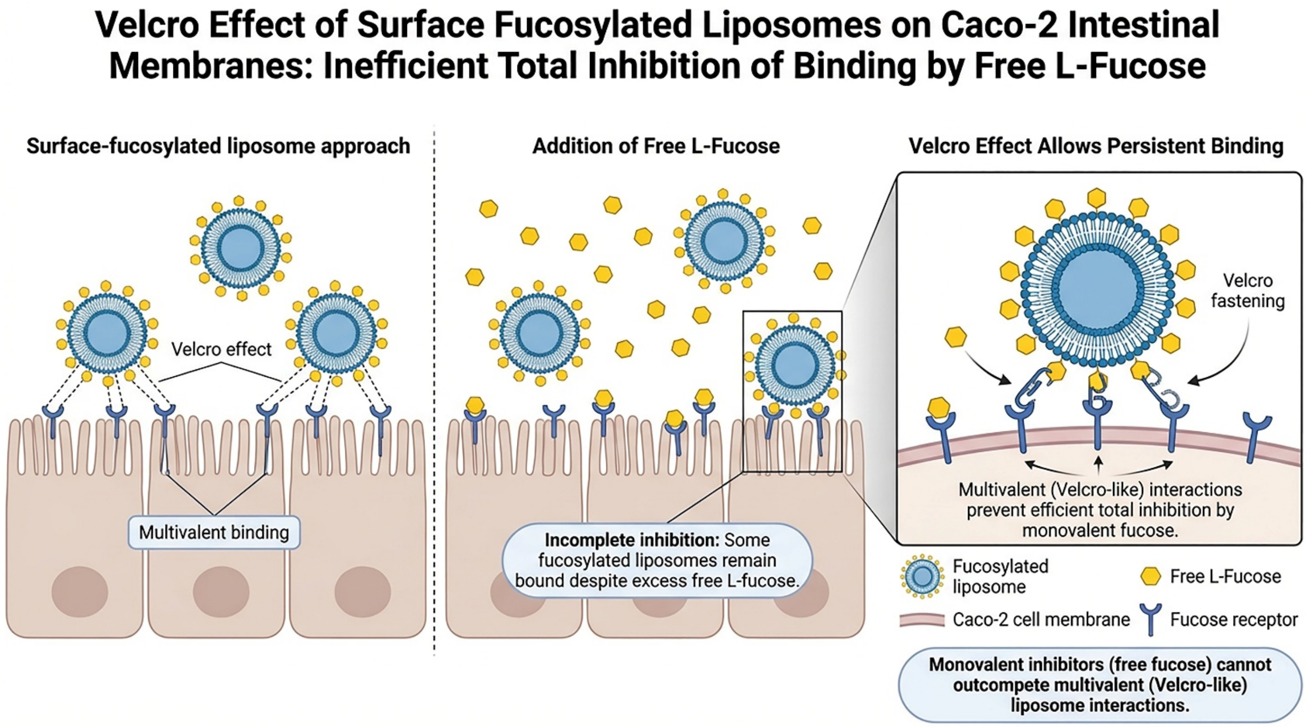

Supplementary Figure 5: The "Velcro Effect" in Multivalent Binding of Fucosylated Liposomes to epithelial intestinal membranes. The schematic illustrates the robust adhesion of surface-fucosylated liposomes to Caco-2 cell receptors via multivalent interactions. Even in the presence of excess free L-fucose (monovalent inhibitor), total inhibition of binding is not achieved. This "Velcro effect" demonstrates that monovalent inhibitors cannot effectively outcompete the high-avidity, multivalent engagement of the liposomal platform, ensuring persistent membrane binding. Figure generated using FigureLabs (figurelabs.ai)

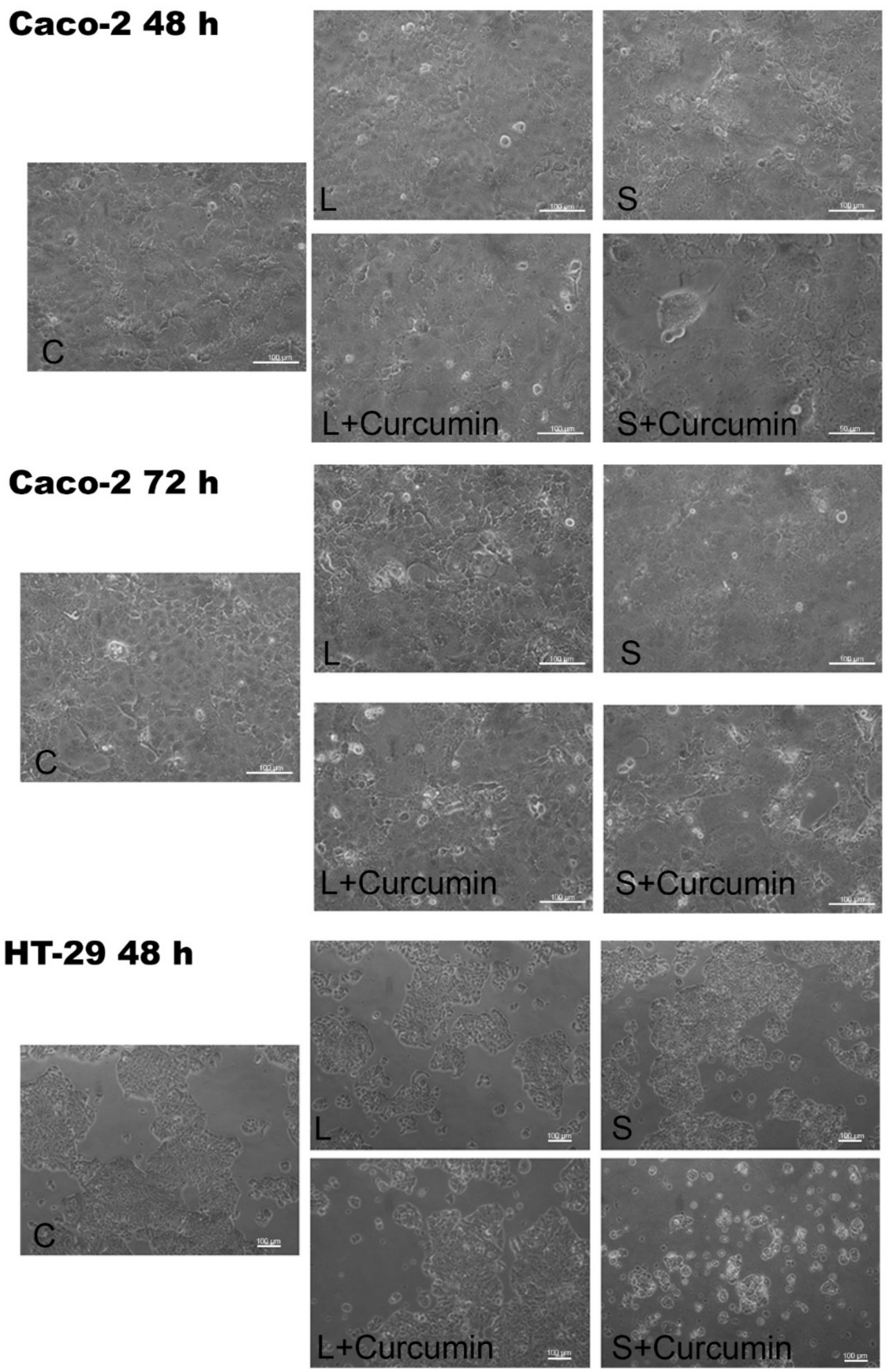

Supplementary Figure 6: Images of brightfield microscopy of Caco-2 treated cells after 48 h and 72 h; and HT-29 treated cells after 48 h. Micrograph are for all different conditions (CTRL, L, L+curcumin, S, S+curcumin) as indicated.

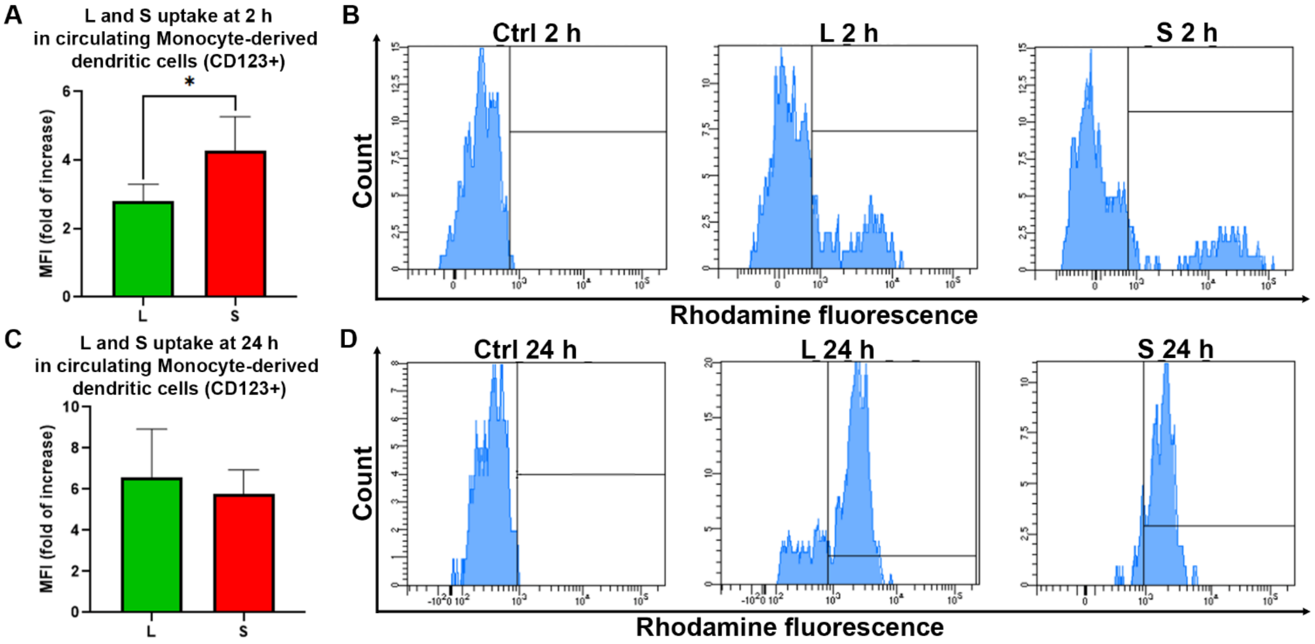

Supplementary Figure 7: Uptake of L and S formulations by circulating monocyte-derived dendritic cells (moDCs). (A) Quantitative analysis of the mean fluorescence intensity (MFI) of L (green) and S (red) formulations in CD123+ moDCs after 2 h. (B) Representative flow cytometry histograms showing the uptake levels in CD123+ moDCs of L, and S-treated in respect of the untreated Control (Ctrl) at 2 h. (C) Quantitative analysis of the mean fluorescence intensity (MFI) of L (green) and S (red) variants in CD123+ moDCs after 24 h. (D) Representative flow cytometry histograms showing the uptake levels in CD123+ moDCs of L, and S-treated in respect of the untreated Control (Ctrl) at 24 h.

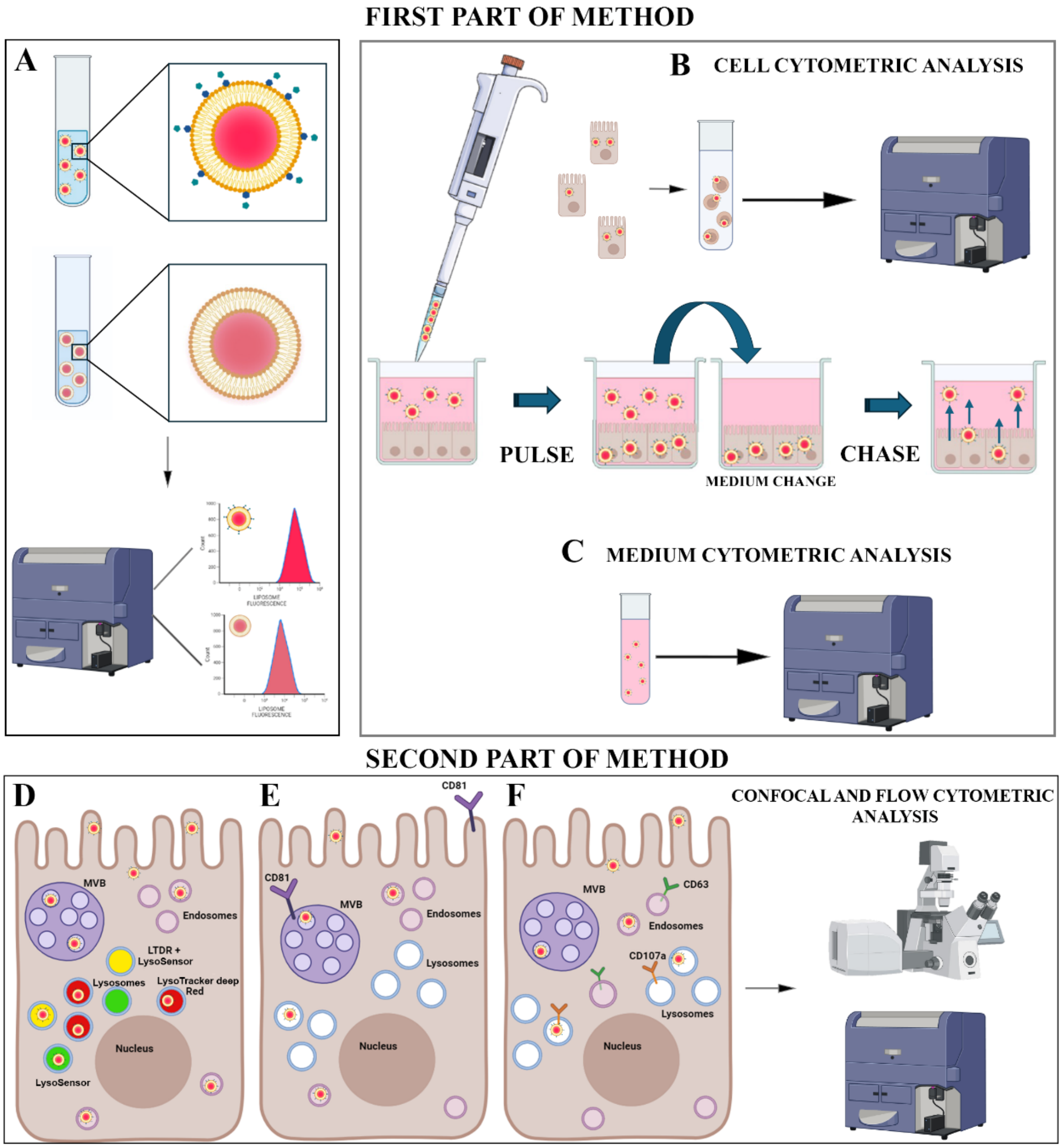

Supplementary Figure 8: Scheme of the flow cytometric tests and confocal analyses representing a cost-effective protocol applicable to monitor uptake, exit processes and intracellular trafficking of drug delivery systems (DDSs). (A) Cytometric analysis of rhodamine-labeled L and S formulations. (B) Pulse and chase experiment to analyze cellular uptake and permanence. (C) Pulse and chase experiment to investigate the release of L and S into the culture medium. (D) Confocal and FC analysis of LysoSensor and LTDR for the evaluation of endosomal escape. (E) Confocal and FC analysis of intracytoplasmic CD81. (F) Confocal and FC analysis of intracytoplasmic CD63 and LAMP1/CD107a.
